# Adaptation and Validation of the International Pelvic Pain Society's Quality of Life Questionnaire in Portuguese

**DOI:** 10.1055/s-0043-1772591

**Published:** 2023-11-09

**Authors:** Letícia Ferracini Lenharo Hayashi, Paulo Augusto Ayroza Galvão Ribeiro, Júlio Cesar Rosa e Silva, Luiz Gustavo Oliveira Brito, Helizabet Salomão Abdalla Ayroza Ribeiro

**Affiliations:** 1 Department of Obstetrics and Gynecology, Faculdade de Medicina de Catanduva, Catanduva, SP, Brazil.; 2 Faculty of Medical Sciences, Santa Casa de São Paulo, São Paulo, SP, Brazil.; 3 Department of Gynecology and Obstetrics, Faculdade de Medicina de Ribeirão Preto, Universidade de São Paulo, Ribeirão Preto, SP, Brazil.; 4 Department of Obstetrics and Gynecology, Faculdade de Ciências Médicas, Universidade de Campinas, Campinas, SP, Brazil.

**Keywords:** surveys and questionnaires, quality of life, translation, validation study, pelvic pain, chronic pain, inquéritos e questionários, qualidade de vida, tradução, estudo de validação, dor pélvica, dor crônica

## Abstract

**Objective** In the present study, our aim was to translate, adapt, and validate the Pelvic Health History Form (a quality of life [QoL] questionnaire) of the International Pelvic Pain Society (IPPS) from English to Portuguese.

**Methods** The study was approved by the Ethics and Research Committee (CEP, in the Portuguese acronym) and the IPPS. The "Transcultural Adaptation" method comprised 5 stages: translation, synthesis, backtranslation, expert review, and pretest. Cultural adaptation and validation included cognitive interviews and statistical analysis of unanswered items (> 15%) in 14 clinic patients from CPP and endometriosis clinic at Santa Casa de São Paulo.

**Results** Strong equivalences were established between the USA and Brazil questionnaires in terms of semantics, idioms, experiences, and concepts. Eighteen culturally inappropriate items were identified and adjusted using the revised response rate index. The subjective form underwent rigorous assessments, confirming its accurate measurement of intended targets.

**Conclusion** The methodology showed efficiency and equivalence, confirming its validity. The user-friendly format and inclusion of translated, adapted, and validated instruments in Portuguese make the form valuable for evaluating pelvic health, with potential for future research.

## Resumo

**Objetivo** Realizar a tradução, adaptação e validação do questionário de qualidade de vida Pelvic Health History Form da International Pelvic Pain Society (IPPS, na sigla em inglês) para a língua portuguesa.

**Métodos** Aprovação do Comitê de Ética e Pesquisa (CEP) e consentimento do IPPS. A metodologia “Adaptação Transcultural” foi utilizada em cinco etapas: (I) tradução; (II) síntese; (III) retradução; (IV) revisão pelo comitê de especialistas; (V) pré-teste, seguido de adaptação cultural e validação por meio de entrevista cognitiva e análise estatística da taxa de ausência de respostas > 15% após aplicação do instrumento em 14 pacientes do ambulatório de DPC e endometriose da Santa Casa de São Paulo.

**Resultados** Equivalências semântica, idiomática, experiencial e conceitual entre o questionário de país fonte (EUA) e alvo (Brasil) foram bem estabelecidas. Dezoito itens culturalmente impróprios, de acordo com o índice de ausência de respostas revisados, adaptados e realizada validade de face e de constructo, avaliando forma subjetiva, confiável que o instrumento mede o que pretende medir.

**Conclusão** A metodologia utilizada foi eficiente, com boa equivalência com o material de origem concluindo a sua validade. Formulário de formato simples, fácil aplicação e compreensão, composto por diversos instrumentos já traduzidos, adaptados e validados em nossa língua. O formulário auxilia avaliação multidimensional da saúde pélvica destas pacientes e poderá ser utilizado em estudos futuros.

## Introduction

Chronic pelvic pain (CPP) is a complex and multifactorial condition, defined by the American College of Obstetricians and Gynecologist as pain in the pelvic area that lasts for ≥ 6 months, is intermittent or constant, cyclic or noncyclic, and strong enough to cause functional disturbances or require medical care. 1 2 It is a health problem estimated to affect between 6 and 27% of women worldwide. 3 This disease is responsible for 20% of gynecological consultations, and 45% of reduced productivity at work leading to significant socioeconomic expenses and rates of up to 66% of association with anxiety and 63% with depression. 4 5 6 Its etiology is quite complex due to the interaction between gynecological, gastrointestinal, urological, musculoskeletal, and psychological disorders with potential mechanisms that lead to sensitization of the nervous system. 7 It is estimated that only 30% of the etiologies attributed to CPP are primarily gynecological, 2 with endometriosis as the main cause, with a rate of 24 to 40%. 8 However, CPP of unknown etiology accounts for up to 55% of cases. 5 Additionally, given the variable, multifactorial, and uncertain nature of the etiology of this syndrome, the difficulty in accurately diagnosing it, and considering CPP a condition with symptoms that are associated with behavioral, affective, social, and cognitive consequences, it is of utmost importance to use a specific questionnaire that addresses these domains and is aimed at comprehensively evaluating all aspects related to CPP. 5 9

The process of translating, adapting and cross-culturally validating a health questionnaire to a new language, culture, or country, requires a unique method that maintains equivalence between the original and the new version. 10 11 Recent reviews in the literature suggest that questionnaire translations should be conducted through guidelines to ensure cultural and linguistic quality. However, current guidelines focus exclusively on the translation and adaptation process, which transforms the questionnaire, but does not cover the validation process that assesses the quality of the questionnaire, which is considered a separate methodological process although both seem to be conjoined. 12

Moreover, Beaton et al. suggest a cross-cultural adaptation guideline based on the first step of the three stages of the process adopted by the Society for Quality of Life Assessment (IQOLA).These guidelines follow six phases that are very similar among most authors: I – initial translation; II – synthesis of the translation; III – backtranslation; IV – expert committee review; V – test of the prefinal version; VI –submission of the document to the developers or coordination committee for appreciation of the adaptation process. 11

The validation stage of instrument formation tends to be more contested by reviewers. Several instruments for measuring this validation process were created; however, they were considered somewhat confusing and ended up being based on an empirical demonstration of the adaptation of this instrument in the target population. 10

Considering the complex nature of CPP, the importance of a precise diagnosis, and the diverse multinational and multicultural characteristics of quality of life questionnaires in their original languages, as well as the necessity to adapt these measures for Brazilian research projects, we undertook the translation, adaptation, and validation process of the Pelvic Health History Form, a quality of life questionnaire revised in June 2019 by the International Pelvic Pain Society (IPPS), specifically for the Portuguese language, aimed at facilitating the diagnosis of CPP within our settings, which lack dedicated questionnaires available in Portuguese.

The objective of the present study was to carry out the translation, adaptation, and validation of the quality of life questionnaire Pelvic Health History Form of the IPPS into Portuguese.

## Methods

A protocol was developed for the translation and validation of the QoL Pelvic Health History Form questionnaire from the IPPS into the Portuguese language. The flowchart outlines the steps involved in this process, with the original English version revised in June 2019 serving as the foundation. Upon submission and approval of the project by the Investigation Review Board (IRB) under CAAE 40166520.8.0000.5479, and with consent from the IPPS, the methodology of "Cross-Cultural Adaptation" was employed following six detailed phases as described below. 10 11 ( Flowchart 1 ).

I – Initial translation: the instrument was translated into Portuguese by a bilingual Brazilian translator with an American Translator Association (ATA) English to Portuguese Certification #464880 who worked independently and had no understanding regarding the purpose of the study, generating the Portuguese version (T1).
II – Translation synthesis: the document was then reviewed by a specialist in the field of chronic pelvic pain and gynecology (Ribeiro H. S. A. A.) who was aware of the objective of the study and made her considerations based on her knowledge of the subject in question.
III – Backtranslation: the revised version was backtranslated into English by two other native Portuguese-speaking bilingual individuals, one of them mastering the theme and object of the study in question and the other non-mastering, generating two new versions in English (BT1 and BT2).
IV – Expert Committee: in the prefinal phase, a committee of experts formed by two gynecologists with expertise in CPP (Ribeiro H. S. A. A. and Silva J. C. R.), a physiotherapist (RFC) and two bilingual translators (Brito L. G. O. and Ribeiro P. A. A. G.) compared all versions (T1, BT1, and BT2) with the original in English. Necessary adjustments and adaptations were made to obtain the appropriate prefinal version of the questionnaire in Portuguese.
V – Testing of the Prefinal version: this version of the questionnaire was applied in a self-test format to a sample of 14 women, randomly selected at the Gynecological Endoscopy and Endometriosis Division of Santa Casa de Misericórdia de São Paulo from June 22 to June 29, 2022. Inclusion criteria were patients with complaints of pelvic pain for > 6 months, aged between 18 and 45 years old, sexually active, and still in menacme. Exclusion criteria included menopausal patients, prior hysterectomy patients, and patients previously treated for pain with medication or surgery patients.

**Flowchart 1:**
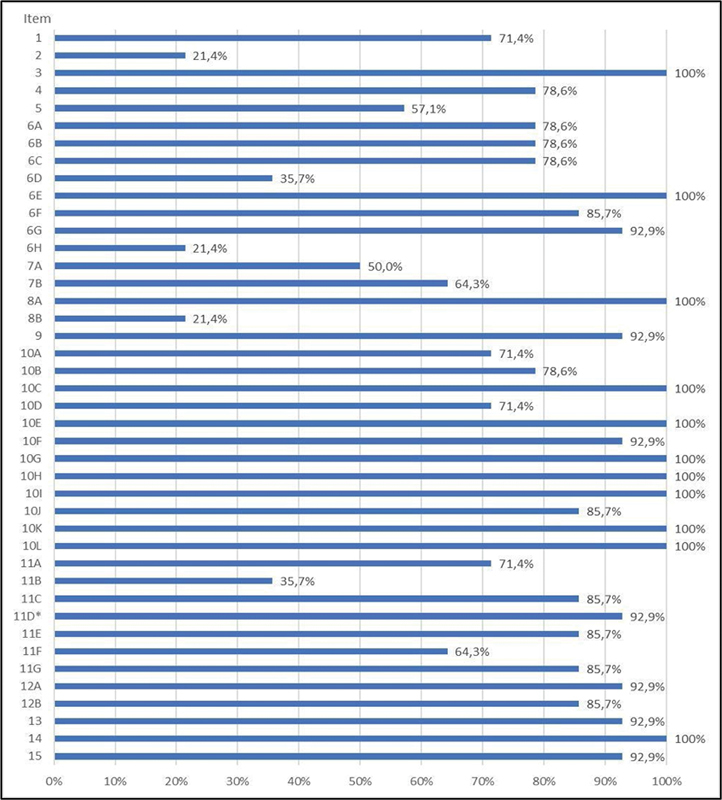
Detailed phases of the process.

To assess cultural adaptation and validation, each patient was led to a private room and invited to participate in the study. Patients were explained the purpose of the questionnaire followed by the signing of the informed consent form. The test was applied individually so that the patient herself answered the questionnaire without help. They were instructed to leave the topics they did not understand unanswered. Immediately after each application, semistructured interviews were carried out for a careful analysis of each item, including their responses addressing their understandings and difficulties in interpretation and responses during the previous phase in order to evaluate the semantic, idiomatic, conceptual, and experiential equivalence with the committee of experts. For statistical purposes, positive responses were considered when the patients had a good reaction to the question.

After this step, a statistical analysis of the response rates for each item and subitem of the questionnaire was performed. 12 13 We evaluated the rate of nonresponses in each item and subitems in order to identify which questions would be culturally incompatible (defined as those with ≥ 15% of nonresponses), so that they could be adapted appropriately. These questions were selected and reviewed by the expert committee to assess the increased rates of nonresponses, focusing on adapting them culturally, while also preserving the same concept in a manner that the structure and evaluation properties of these questions were not altered. After conducting a comprehensive analysis, the committee of translation experts engaged in insightful discussions to identify areas for enhancement and implement necessary adaptations to the questionnaire. As a result, the final version was successfully completed, ensuring semantic, grammatical, experiential, and conceptual equivalence of the QoL questionnaire in Portuguese. The validated instrument was then submitted to the IPPS for approval and recognition ( Supplementary Material Annex 1 ).

## Results

The demographic profile of the sample, as presented in Table 1 , indicated that the patients had a mean age of 34.5 years old (standard deviation [SD]: 8.2), the majority of patients self-identified as white (42.9%) and were married (42.9%), half of the patients reported being sexually active with men (50%), ∼ 70% of the sample had completed high school and/or higher education, more than half of the participants (57.1%) were employed outside the home, and an equal percentage (57.1%) lived with a partner.

**Table 1:** Demographic dada

	*n*	%	Mediam/SD/Min-Max
Age (years old)	12	–	34,5 (8,2) (21-44)
Ethnicity	–	–	–
White	6	42.9	–
Black	5	35.7	–
Other	3	21.4	–
Marital status	–	–	–
Single	2	14.3	–
Married	6	42.9	–
Widowed	1	7.1	–
Stable relationship	4	28.6	–
No answer	1	7.1	–
Sexual practice			–
Sexually active with men	7	50.0	–
Sexually active with women	3	21.4	–
Abstinent	3	21.4	–
No answer	1	7.1	–
Level of Education			–
< 12 years of study	2	14.3	–
Complete high school	5	35.7	–
Complete university education	5	35.7	–
No answer	2	14.3	–
Type of work			–
Unemployed	3	21.4	–
Works outside home	8	57.1	–
Homemaker	3	21.4	–
Coexistence			–
Alone	1	7.1	–
Partner	8	57.1	–
Relatives	3	21.4	–
Another family member	2	14.3	–

Abbreviation: SD, standard deviation.

Following the administration of the instrument and conducting individual interviews for questionnaire analysis, we obtained positive and satisfactory results. Most patients showed good acceptance of the instrument as indicated by their favorable evaluation of the concepts addressed. These findings are summarized in Table 2 , which was created based on the analysis of the questions asked during the semistructured cognitive interviews.

**Table 2:** Evaluation of positive responses per item n (%) of patients with chronic pelvic pain at the gynecological endoscopy and endometriosis outpatient clinic

Question	*n* (%)
1. Does the questionnaire represent the pain you feel?	14 (100%)
2. Has everything you feel been addressed? Or was something missing?	14 (100%)
3. Do you believe that the instrument will help health professionals to better understand your pain?	14 (100%)
4. Did you understand everything that was asked? If not, what did you not understand?	9 (64.3%)
5. Did you have difficulty with any answers?	3 (21.4%)
6. Did you have difficulty with any specific words or interpretations?	13 (92.9%)
7. Were you embarrassed to answer?	14 (100%)
8. Did you find it time consuming? And how long do you think it took to respond?	4 (28.6%)
9. Do you think the questionnaire could be shortened?	8 (57.1%)
10. What did you think of the questionnaire? Do you believe it will be useful in helping your diagnosis?	14 (100%)

Among the questions that did not receive 100% positive responses, we noticed that in item 4, which asked if patients understood everything they were asked, the majority (64.3%) understood most of the questionnaire. However, some patients highlighted flaws and left certain questions blank (items 11 and 12) due to confusion caused by the translated image tables. Regarding item 5, which asked if there were difficulties with any response, 21.4% of the participants experienced difficulties with the response format for item 11, which was based on the McGill pain quality approach. In item 6, which inquired about difficulties with specific words or interpretations, 7.1% of the participants had trouble understanding the word *vesicais* (item 14), which addressed *infecções urinárias frequentes* . Moreover, these observations provide valuable feedback on areas that may require further clarification or refinement in the translated instrument. Regarding the time to complete the form, when asked in question 8 if they found it time-consuming and how long they estimated it took to answer, only 35.7% of the patients did not consider it time-consuming, while 28.6% thought it was time consuming, and 35.7% thought it was just a bit time consuming. Overall, patients were dissatisfied with the time it took to complete the questionnaire. The perception of time varied from 10 to 60 minutes (median of 32.5 minutes), while the reality of scheduled time ranged from 31 to 75 minutes (median of 58.0 minutes). The comparison between the perceived time and the actual scheduled time was assessed using the p-value obtained from the Wilcoxon test, as presented in Table 3 .

**Table 3:** Assessment of perception of time in minutes by patients with chronic pelvic pain

	*n*	median (min–max)	*p-value*
Perception	12	32.5 (10–60)	< 0.001 **
Real time	13	58.0 (31–75)	

** p-value obtained by the Wilcoxon test.

**Fig. 1:**
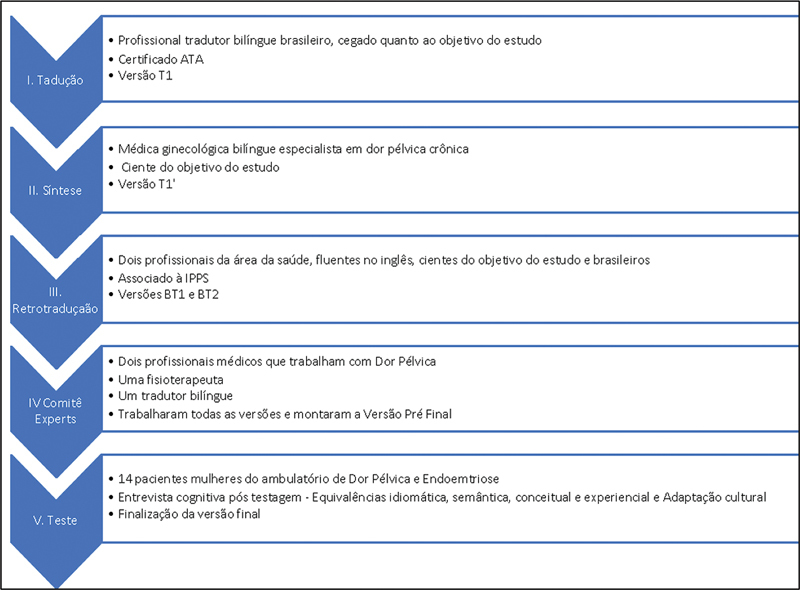
Percentage of responses per item.

A little over half of the participants (57.1%) expressed that, despite its length, the questionnaire should not be shortened. They believed that all the questions included were important, which was evident when asked if they thought the questionnaire could be shortened. During the statistical analysis of the questionnaire responses, we examined a total of 42 items/subitems. Among these, we identified 18 that were deemed culturally inappropriate, based on the criterion of response absence rate > 15%. These findings are depicted in Fig. 1 .

## Discussion

Our study was inspired by the understanding of the complexity of CPP and the realization that there is no extensive questionnaire available that could guide us more easily towards the diagnosis of the disease. Therefore, we chose to carry out the translation, adaptation, and validation into Portuguese of the quality of life questionnaire Pelvic Health History Form of the International Pelvic Pain Society revised in June 2019.

The process of cross-cultural adaptation and validation is indeed lengthy, but it holds significant importance in adapting multinational/cultural quality questionnaires and promoting the use of these measurements in Brazilian research projects.

As the literature suggests, the guidelines used in the questionnaire translation process were based on the cross-cultural adaptation of the medical, sociological, psychological, and methodological literature, resulting in a complete adaptation process that conferred a semantic, idiomatic, experiential, and conceptual equivalence between the source country (USA) and target country (Brazil) questionnaires, as well with several other already recognized questionnaires. 10 11 12 14 15 16 17 18 19

The backtranslations demonstrated a high level of agreement with the original English version. Cognitive interviews conducted with semistructured questions indicated excellent acceptability and comprehension of the questionnaire, consistent with previous findings by other researchers ( Table 2 ). 18 19 All respondents regarded the items as relevant (questions 1, 2, and 3), non-offensive (question 7), and useful (question 10). Approximately 64% of the patients found the questions to be easily understandable (question 4), and there were minimal difficulties encountered regarding specific terminology or the interpretation of questions and answers (questions 5 and 6). None of the participants suggested any modifications to the instrument, although over half of them (64.3%) acknowledged that the questionnaire could be perceived as “time-consuming” or “slightly time-consuming"; however, they did not think it should be reduced (57%).

An important consideration is the method of questionnaire administration. In the literature, we observed that the SF-36 questionnaire was designed to be a straightforward instrument, with clear and easily understandable direct questions, typically administered in a self-report format. A crucial characteristic of a questionnaire is its reasonable application time. In comparison with the translation and validation study of the SF-36, which reported an average application time of 7 minutes, our study recorded an average time of 58 minutes. This discrepancy may explain the higher rate of unanswered questions as the patients did not exhibit difficulties with the questions during the interviews themselves.

The translated questionnaire utilized simple language, making it easy to understand and easy to self-administer. Although a few items analyzed showed a higher rate of unanswered response (above or equal to the acceptable threshold of 15%), primarily in questions related to contact information (item 1), professional contact information (item 2), clinical history (item 4) and surgical history (item 5), menstrual history, birth control, and STIs (items 6A, 6B, 6C, 6D, and 6H), allergies and medications (items 7A and 7B), obstetric history (8B), history, description, and contributing factors of pain (10A , 10B, and 10D), location of pain, intensity scales and treatments performed (11A, 11B, 11D, and 11F), most of these instances were due to a lack of attention, often precipitated by the lengthy completion time of the questionnaire. As identified through the cognitive interview, there was no need to modify the content of the questions or answers, hence only minor text formatting corrections were made.

During the cognitive interview, a comprehensive analysis of all items in the translated questionnaire was conducted, comparing the findings with the completion statistics. The most significant observations were carefully examined and discussed. Regarding item 1, which focused on contact information, although all participants indicated their understanding of the question, many were reluctant to provide personal identification, even though they were assured of data confidentiality. This justified the slightly higher response rate, which remained above the recommended threshold of 15%. Additionally, in this item, it was decided to adapt the response options by including "Portuguese" as an answer choice for preferred language of communication.

Furthermore, in item 6D, where participants were asked about the duration of their menstrual cramps, it was found that 64.3% did not provide a response. Many participants explained that they experienced pain but not specifically cramps. Therefore, it was determined to modify the term to "menstrual pain" instead of "menstrual cramps" to better align with participants' experiences. Regarding the item addressing ethnicity (item 3), although it was considered culturally appropriate based on the response rate, it was decided to adapt it according to the race classification defined by the Brazilian Institute of Geography and Statistics (IBGE, in the Portuguese acronym) to ensure consistency with the categorization of the population. These adaptations and modifications were made to enhance the clarity, relevance, and cultural appropriateness of the questionnaire based on the cognitive interview feedback.

The study findings indicated that the utilization of simple, objective, and direct questions with minimal descriptors was associated with a lower rate of unanswered responses. Notably, items 8A, 10E, 10G, 10H, 10I, 10K, and 10L achieved a complete response rate of 100% without any reported difficulties by the participants

The assessment of pain location, intensity, and characteristics, as well as psychological aspects, were based on questionnaires already recognized, translated, and validated into Portuguese. These included the short version of the McGill Questionnaire, the Visual Analogue Scale (VAS), the short form of the Pain Intensity Scale, the Sexual Function Profile PROMIS version for women, men, and global health, and the Pain Catastrophizing Scale (PCS) and the DASS-2.

Regarding the characteristics of pain in the short version of the McGill questionnaire contained in item 11B, 9 patients (64.3%) did not provide a response. This observation raises several considerations. Notably, the patients encountered the greatest difficulty in answering this particular item, likely due to the complex evaluation format employed in the original proposed form. In addition, the example formatting was less prominent, and there was a formatting error in the answer table, which led to confusion and subsequent difficulty in understanding and responding. In comparison, the other scales mentioned in this form were found to have clearer questions and answers, resulting in a higher response rate (items 11C/D/E and 15). However, since there was no semantic and conceptual difficulty, it was decided to keep the translation according to the original McGill content and highlight only its presentation form to facilitate understanding. The McGill Questionnaire (MPQ) was initially developed in 1970 and has since been considered as an important tool for the quantitative assessment of subjective pain experiences. Its shorter version was developed a decade later (Sort Form-MPQ), consisting of 15 descriptors (11 sensory and 4 affective) and complemented with the VAS and the verbal scale of pain in the present moment (present pain intensity [PPI]). In our questionnaire, derived from this instrument, we maintained the anatomical map from the original version, along with the 15 descriptors and their qualitatively classified intensities (mild, moderate, and severe). Additionally, items were included to assess the duration of the pain, the guided location using the anatomical map, and the VAS. The purpose of this item is to evaluate the location, duration, type, and qualitative intensity of pain. However, the formatting of this item was found to be generalized and visually complex, leading to some confusion during its completion, even among the expert professionals who assisted in validating the questionnaire. Despite this issue, it was determined that the official translation provided by Menezes Costa et al. in 2011, which had already undergone validation, would be retained for consistency. 20 21

As mentioned in the translation and validation study conducted by Flynn et al. in 2013, the subitem 11D of our questionnaire includes the SexFS Version 1.0 from PROMIS. This questionnaire was specifically designed to assess sexual function and satisfaction in both males and females, comprising a total of 81 items. 22 The psychometric properties of this instrument were evaluated using both quantitative (development scales) and qualitative (psychometric evaluation) measures, providing researchers with information regarding its reliability and validity as a measure of sexual function and satisfaction for both genders. However, it is worth noting that a criticism of this study is the lack of testing the male version on male participants. The Global Health Scale v1.1, developed by PROMIS, is notable for its ability to assess overall perceptions of physical, mental, and social health using a limited number of items. This scale consists of a total of 10 items and can be completed in a short time, ∼ 2 minutes. The scale has been previously translated and validated by Zumpano et al. in 2017. In our questionnaire, the eight items used for scoring are included from this instrument, as well as the item Global 07 from the original scale. However, there are no fields or cutoff points provided for evaluating the results. It should be noted that the global nonscoring question 08, which asks about fatigue, is absent in our version, which may be considered a limitation of the original IPPS questionnaire. 23 The DASS-21 questionnaire, a shortened version of the DASS questionnaire, is included in our questionnaire as item 15. It consists of three subscales, each containing seven items that assess emotional aspects such as depression, anxiety, and stress. The questionnaire consists of a total of 21 items that are rated on a 4-point Likert scale. The translation and adaptation of the DASS-21 to Brazilian Portuguese was conducted by Vignola et al. in 2014, 24 following a methodology similar to the cross-cultural method used in our study. The translation process involved multiple iterations of translation, revision, backtranslation, and finalization. It takes approximately 30 minutes to complete the DASS-21 questionnaire.

It should be noted that in the original IPPS questionnaire, the last item of the DASS-21 questionnaire was replicated, while the last item of the original DASS-21 version contains a different statement. This discrepancy may indicate a potential flaw in the original version of the questionnaire. It was observed that, despite all these QoL questionnaires having their scales and classification scores already predefined, none of these questionnaires (items 11A/D/E and 15) contained in the form provide fields for calculating points, scores, or reference cutoffs to evaluate the results.

During the review of the completed questionnaires, it was noticed that several items, although considered completed, were incomplete, with only the affirmative answer “yes” marked, while the negative response "no" was ignored when the participant did not identify with the question (items 5, 11F, 13, and part of 14) in contrast to Cicconelli, 1997, who suggests that a questionnaire should preferably be presented in a simple format and language, being easy to apply and understand. 15 This may indicate that the extensive format of the form, possibly leading to fatigue, despite being well accepted, and the inclusion of multiple questionnaires within it contribute to the increased completion time.

To assess the acceptability and understanding of the questionnaire, similar to the present study, Hasvik et al. and Zumpano et al. also conducted semistructured cognitive interviews regarding the general meaning of the questionnaire, the explanatory text, the pain descriptors, and the answers (numerical classification). 19 23 The number of patients tested with the prefinal version was also quite variable, ranging from just 5 to even using 100 patients in the surveyed literature. 16 23

Systematic reviews on the methodological studies related to the development and validation of questionnaires have concluded that there is no established set of criteria to determine the quality of property measures. The literature in this field is highly variable, often relying on expert opinions, even if based on literature reviews. 12 25

These reviews suggest that if the content validity of a questionnaire is adequate, it can be considered usable, and it is sufficient to test the translated instrument against the original questionnaire for validation purposes. In the case of this questionnaire, which includes several previously translated and validated instruments (SF-McGill, PCS, VAS, PROMIS sexual function, global health, and DASS-21) in Portuguese, we can consider it validated. Therefore, there was no evaluation of the psychometric properties conducted in the present study. However, it is important to acknowledge that this may be considered a weakness of the instrument.

Cicconelli (1997) emphasizes the importance of reproducibility in evaluation instruments, requiring consistent results across multiple administrations for the same patient, assuming their general clinical state remains unchanged. In the present study, the evaluation of reproducibility was not conducted since the questionnaire serves as an identification form and initial anamnesis, rather than a comprehensive instrument intended for repeated administration in the same patients. Furthermore, Cicconelli emphasizes that a measure is considered valid when it effectively measures what it intends to measure. Considering the systematic review by Terwee, which highlights content validity as a crucial property measure, and the absence of a gold standard instrument for criterion validity comparison, face and content validity were thoroughly assessed using a subjective and reliable format, ultimately consolidating the validity of this instrument.

The Pelvic Health History Form in Portuguese will significantly contribute to the diagnosis and appropriate treatment of CPP, leading to improved quality of life for women with CPP. Moreover, its availability will foster research endeavors in Brazil.

## Conclusion

The translation and cross-cultural adaptation process demonstrated exceptional efficiency, achieving a high level of equivalence with the source material while preserving both qualitative and quantitative validity. The resulting questionnaire is presented in a user-friendly format, ensuring ease of application and comprehension. This significant development represents a notable stride forward and its growing presence in the literature, particularly for self-administration in clinical settings while establishing a strong foundation for future studies to build upon.

## References

[1] Pain C P ACOG Practice Bulletin. Number 218Obstet Gynecol 2020 3 e98 e109 10.1097/AOG.0000000000003716 32080051

[2] Carey E T Till S R As-Sanie S Pharmacological management of chronic pelvic pain in women Drugs 2017 77 03 285 301 28074359 10.1007/s40265-016-0687-8

[3] Ahangari A Prevalence of chronic pelvic pain among women: an updated review Pain Physician 2014 17 02 E141 E147 24658485

[4] Lewis S C Bhattacharya S Wu O Vincent K Jack S A Critchley H OD Gabapentin for the management of chronic pelvic pain in women (GaPP1): a pilot randomised controlled trial PLoS One 2016 11 04 e0153037 27070434 10.1371/journal.pone.0153037 PMC4829183

[5] Chong O T Critchley H O Horne A W Fallon M Haraldsdottir E Chronic pelvic pain in women: an embedded qualitative study to evaluate the perceived benefits of the meridian balance method electro-acupuncture treatment, health consultation and National Health Service standard care Br J Pain 2019 13 04 244 255 31656631 10.1177/2049463718814870 PMC6791054

[6] Siqueira-Campos VMe Da Luz R A de Deus J M Martinez E Z Conde D M Anxiety and depression in women with and without chronic pelvic pain: prevalence and associated factors J Pain Res 2019 12 1223 1233 31114304 10.2147/JPR.S195317 PMC6490234

[7] Allaire C Williams C Bodmer-Roy S Zhu S Arion K Ambacher K Chronic pelvic pain in an interdisciplinary setting: 1-year prospective cohort Am J Obstet Gynecol 2018 218 01 1140 1.14E14 10.1016/j.ajog.2017.10.002 29031895

[8] Armour M Lawson K Wood A Smith C A Abbott J The cost of illness and economic burden of endometriosis and chronic pelvic pain in Australia: A national online survey PLoS One 2019 14 10 e0223316 31600241 10.1371/journal.pone.0223316 PMC6786587

[9] Santosh A Liaquat H B Fatima N Liaquat S Anwar M A Chronic pelvic pain: a dilemma J Pak Med Assoc 2010 60 04 257 260 20419964

[10] Guillemin F Cross-cultural adaptation and validation of health status measures Scand J Rheumatol 1995 24 02 61 63 7747144 10.3109/03009749509099285

[11] Beaton D E Bombardier C Guillemin F Ferraz M B Guidelines for the process of cross-cultural adaptation of self-report measures Spine 2000 25 24 3186 3191 11124735 10.1097/00007632-200012150-00014

[12] Danielsen A K Pommergaard H-C Burcharth J Angenete E Rosenberg J Translation of questionnaires measuring health related quality of life is not standardized: a literature based research study PLoS One 2015 10 05 e0127050 25965447 10.1371/journal.pone.0127050 PMC4428794

[13] Ciconelli R M Ferraz M B Santos W Meinão I Quaresma M R Tradução para a língua portuguesa e validação do questionário genérico de avaliação de qualidade de vida SF-36 (Brasil SF-36) Rev Bras Reumatol 1999 39 03 143 150

[14] Melzack R The McGill Pain Questionnaire: major properties and scoring methods Pain 1975 1 03 277 299 1235985 10.1016/0304-3959(75)90044-5

[15] VALIDAÇÃO TPOPE. ROZANA MESQUITA CICONELLI: Escola Paulista de Medicina;1997

[16] Apóstolo J L Mendes A C Azeredo Z A Adaptation to Portuguese of the Depression, Anxiety and Stress Scales (DASS) Rev Lat Am Enfermagem 2006 14 06 863 871 17294019 10.1590/s0104-11692006000600006

[17] Sehn F Chachamovich E Vidor L P Dall-Agnol L Souza I CC Torres I LS Cross-cultural adaptation and validation of the Brazilian Portuguese version of the pain catastrophizing scale Pain Med 2012 13 11 1425 1435 23036076 10.1111/j.1526-4637.2012.01492.x

[18] Cavalcante J A Viana K A Costa P S Costa L R Tradução, adaptação transcultural e avaliação preliminar da pain catastrophizing scale-parents para uso no brasil Rev Paul Pediatr 2018 36 428 436 30540108 10.1590/1984-0462/;2018;36;4;00014 PMC6322810

[19] Hasvik E Haugen A J Haukeland-Parker S Rimehaug S A Gjerstad J Grøvle L Cross-cultural adaptation and validation of the Norwegian short-form McGill pain questionnaire-2 in low back-related leg pain Spine 2019 44 13 E774 E781 31205173 10.1097/BRS.0000000000002976

[20] Melzack R The short-form McGill pain questionnaire Pain 1987 30 02 191 197 3670870 10.1016/0304-3959(87)91074-8

[21] Menezes Costa LdaC Maher C G McAuley J H Hancock M J Oliveira W M Azevedo D C The Brazilian-Portuguese versions of the McGill Pain Questionnaire were reproducible, valid, and responsive in patients with musculoskeletal pain J Clin Epidemiol 2011 64 08 903 912 21444194 10.1016/j.jclinepi.2010.12.009

[22] Flynn K E Lin L Cyranowski J M Reeve B B Reese J B Jeffery D D Development of the NIH PROMIS ® Sexual Function and Satisfaction measures in patients with cancer J Sex Med 2013 10(0 1, Suppl 1)43 52 23387911 10.1111/j.1743-6109.2012.02995.x PMC3729213

[23] Zumpano C E Mendonça T M Silva C H Correia H Arnold B Pinto R M [Cross-cultural adaptation and validation of the PROMIS Global Health scale in the Portuguese language] Cad Saude Publica 2017 33 01 e00107616 28125122 10.1590/0102-311X00107616

[24] Vignola R CB Tucci A M Adaptation and validation of the depression, anxiety and stress scale (DASS) to Brazilian Portuguese J Affect Disord 2014 155 104 109 24238871 10.1016/j.jad.2013.10.031

[25] Terwee C B Bot S D de Boer M R van der Windt D AWM Knol D L Dekker J Quality criteria were proposed for measurement properties of health status questionnaires J Clin Epidemiol 2007 60 01 34 42 17161752 10.1016/j.jclinepi.2006.03.012

