# Supplementary material for: Adaptation and Validation of the International Pelvic Pain Society's Quality of Life Questionnaire in Portuguese
Source: Rev Bras Ginecol Obstet. 2023 Nov 9;45(10):e575–83. doi: 10.1055/s-0043-1772591 (PMC10635788; doi:10.1055/s-0043-1772591)
Supplement: Supplementary file 1 — Supplementary Material [file 10-1055-s-0043-1772591_s230045.pdf]

Supplementary Material Annex 1. Pelvic health history form

INTERNATIONAL PELVICPAIN SOCIETY

PELVIC HEALTH HISTORY FORM

www.pelvicpain.org

Today's Date: ______ **Chart Number (FOR OFFICE USE ONLY):** ______

1. Contact information

Legal Last Name: ______ **Legal First Name:** ______

Date of Birth: ______ **Age:** ______

Email: ______ **Phone:** ______

How do you prefer to be addressed? ***(Check all that apply)***

☐ She / Her ☐ He/Him ☐ Them/They ☐ Dr. ☐Legal last name ☐ Legal first name

☐Other Name: ______ ☐Other gender pronoun: ______

What language do you prefer to communicate in? ***(Check all that apply)***

☐English ☐Spanish ☐ French ☐Other: ______

2. Referring provider's name and contact information:

Name: ______ **Phone:** ______ **Contact address:** ______

How many doctors or health care providers have you seen in the past for your pelvic pain?

☐None ☐1 ☐2 ☐3 ☐4 ☐5 ☐6 ☐7 ☐8 ☐9 ☐10 ☐>10

3. Demographic information:

What race and ethnicity best describes you? ***(Check all that apply)***

☐American Indian or Alaskan Native ☐Asian ☐Native Hawaiian or Pacific Islander
☐Black or African American ☐White ☐Middle Eastern
☐Hispanic or Latino/a/x ☐Other: ______

What is your relationship status? ***(Check all that apply)***

☐Single ☐Married ☐Separated ☐Divorced ☐Widowed ☐Partnered ☐Casually dating
☐Other: ______

Describe your sexual practices: ***(Check all that apply)***

☐NOT sexually active / abstinent ☐Asexual (without sexual feelings or associations)
☐Sexually active with men ☐Sexually active with women ☐Sexually active with both
☐Other: ______

With whom do you live? ***(Check all that apply)***

☐Alone ☐Partner ☐Parents ☐Other Family ☐Friends ☐Homeless ☐Other: ______

What is your education? ***(Check only one)***

☐Less than 12 years ☐High School graduate ☐College degree ☐ Postgraduate degree

What type of work are you doing? ***(Check only one)***

☐Unemployed ☐Work outside home ☐Homemaker ☐Retired ☐Disabled

Last revised 6.19.2019 info@pelvicpain.org, www.pelvicpain.org

All information, content, and material on this form is for informational purposes only and is not intended to serve as a substitute for the consultation, diagnosis, and/or medical treatment of a qualified physician or healthcare professional.

1

 © 2023. Federação Brasileira de Ginecologia e Obstetrícia. All rights reserved.

INTERNATIONAL PELVICPAIN SOCIETY

PELVIC HEALTH HISTORY FORM

www.pelvicpain.org

4. Medical History

Please list your medical or health problems, describe when the condition was diagnosed and whether it is controlled.

Medical Problem	Year Diagnosed	Controlled?
		Yes☐ No☐
		Yes☐ No☐
		Yes☐ No☐
		Yes☐ No☐
		Yes☐ No☐
		Yes☐ No☐

5. Surgical History

Please check if you have had any of the following surgeries

Procedure		Date	Surgeon	Findings
Cystoscopy (looking inside the bladder)	☐Yes ☐No			
Laparoscopy w/removal of Endometriosis	☐Yes ☐No			
Hysterectomy (removal of uterus and cervix)	☐Yes ☐No			
Were your ovaries removed?	☐Yes ☐No			
Was the cervix retained (Supra-cervical hysterectomy)?	☐Yes ☐No			
Myomectomy	☐Yes ☐No			
Endoscopy	☐Yes ☐No			
Colonoscopy	☐Yes ☐No			
Ovarian Cyst Removal	☐Yes ☐No			
Cesarean Delivery	☐Yes ☐No			
Appendectomy (appendix removal)	☐Yes ☐No			
Prostatectomy	☐Yes ☐No			
Colectomy (removal of colon)	☐Yes ☐No			
Vasectomy	☐Yes ☐No			
Other:				

Last revised 6.19.2019 info@pelvicpain.org. www.pelvicpain.org

All information, content, and material on this form is for informational purposes only and is not intended to serve as a substitute for the consultation, diagnosis, and/or medical treatment of a qualified physician or healthcare professional.

2

 © 2023. Federação Brasileira de Ginecologia e Obstetrícia. All rights reserved.

INTERNATIONAL PELVICPAIN SOCIETY

PELVIC HEALTH HISTORY FORM

www.pelvicpain.org

6. Menstrual, Birth Control and Sexually Transmitted Infections History

If you DO NOT menstruate, select the reason(s) why: ***(Check all that apply)***

☐Had a hysterectomy ☐Menopause ☐ Assigned MALE at birth ***then skip to*** →

☐On continuous menstrual suppression using birth control (e.g. Depoprovera, pills, Progesterone IUD)

☐Had an Endometrial ablation

When was your last menstrual period?

How old were you when your menstrual cycles started?

If you menstruate, do you CURRENTLY have any of the following symptoms DURING menstruation? ***(Check all that apply)***

☐Heavy bleeding ☐Severe pain ☐ Irregular bleeding (more than once a month) ☐Bleeding > 7 days

☐Mood swings ☐Fatigue ☐Breast tenderness ☐Constipation ☐Diarrhea ☐Headaches

If you have painful periods, how long have you had this type of pain? Please specify years or months.

Do you CURRENTLY regularly (more than 3 times a month) miss school or work due to your painful period?

☐Yes ☐No

If you have painful periods, have you used any of the following to help with your pain during your period? ***(Check all that apply)***

☐ Birth Control Pill ☐ Vaginal ring ☐ Depo Provera ☐ Hormonal IUD

☐NSAIDS (e.g. Ibuprofen, Naproxen) ☐Acetaminophen ☐Other:

What are you using for birth control / contraception? ***(Check all that apply)*** ←

☐ Nothing ☐ Vasectomy ☐Condoms ☐ Birth control pills ☐ Depoprovera injection

☐ Nexplanon implant ☐ Vaginal ring (NuvaRing) ☐Tubal Ligation

☐ Hormonal IUD ☐ Non-Hormonal IUD Other:

Have you ever had any sexually transmitted infections (STIs)? ***(Check all that apply)***

☐Chlamydia ☐Gonorrhea ☐Herpes ☐HPV (Human Papilloma Virus) ☐Syphilis

☐PID (Pelvic Inflammatory Disease) ☐HIV ☐Hepatitis B ☐Hepatitis C

7. Allergies and Current Medications

Please list your allergies:

Allergy	Reaction, what happens when you have this allergy?	Have you had treatments in the past for this allergy?

Last revised 6.19.2019 info@pelvicpain.org. www.pelvicpain.org

All information, content, and material on this form is for informational purposes only and is not intended to serve as a substitute for the consultation, diagnosis, and/or medical treatment of a qualified physician or healthcare professional.

3

 © 2023. Federação Brasileira de Ginecologia e Obstetrícia. All rights reserved.

INTERNATIONAL PELVIC PAIN SOCIETY

PELVIC HEALTH HISTORY FORM

www.pelvicpain.org

Please list all CURRENT medications you are taking, including herbal remedies:

Medication or Herbal Remedies	Dose	For what medical condition

8. Pregnancy / Obstetric History

How many pregnancies have you had? ☐0 ☐1 ☐2 ☐3 ☐4 ☐5 ☐6 or more

How many deliveries have you had? ☐0 ☐1 ☐2 ☐3 ☐4 ☐5 ☐6 or more

How many deliveries were vaginal? ☐0 ☐1 ☐2 ☐3 ☐4 ☐5 ☐6 or more

How many deliveries were cesarean? ☐0 ☐1 ☐2 ☐3 ☐4 ☐5 ☐6 or more

How many were miscarriages or abortions? ☐0 ☐1 ☐2 ☐3 ☐4 ☐5 ☐6 or more

Where there any complications during pregnancy, labor, delivery, or postpartum?
☐ Laceration 3°- 4° ☐Vacuum/ Forceps ☐Wound complication ☐Other

9. Family History

Has anyone in your family had any of the following condition(s)? *(Check all that apply)*

☐Endometriosis ☐Fibromyalgia ☐Chronic pelvic pain ☐Irritable bowel syndrome ☐ Interstitial Cystitis
☐Colon Cancer ☐Breast Cancer ☐Uterine Cancer ☐Ovarian Cancer ☐Depression
☐Chronic Fatigue Syndrome ☐ Anxiety/Panic Attacks ☐Temporomandibular Joint Disorder (TMD)
☐Migraine Headache ☐Post-Traumatic Stress Disorder (PTSD)
☐Other Chronic Condition:

Last revised 6.19.2019 info@pelvicpain.org. www.pelvicpain.org

All information, content, and material on this form is for informational purposes only and is not intended to serve as a substitute for the consultation, diagnosis, and/or medical treatment of a qualified physician or healthcare professional.

4

 © 2023. Federação Brasileira de Ginecologia e Obstetrícia. All rights reserved.

INTERNATIONAL PELVICPAIN SOCIETY

PELVIC HEALTH HISTORY FORM

www.pelvicpain.org

10. Pain History, Description and Contributing Factors

When did your pain begin? Month: Year: ☐Unsure

Please use your own words to describe your pain:

How did your main pain begin, do you recall a specific incident that occurred when your pain first began? ***(Check one)***

☐Injury at home ☐Injury at work/school ☐Injury in other setting ☐Motor vehicle crash
☐After surgery ☐Cancer ☐Medical condition other than cancer
☐No obvious cause/ do not know a specific incident ☐Other:

How did your pain begin? ***(Check only one)*** ☐Suddenly ☐Gradually

How long has your main pain been present? ***(Check only one)***

☐Less than 3 months ☐3-12 months ☐12 months-2 years ☐2-5 years ☐More than 5 years

Since your pain began, is your pain: ***(Check only one)***

☐No different ☐Getting better ☐Getting worse ☐I don't know

Which statement best describes your pain? ***(Check only one)***

☐Always present (always the same intensity)
☐Always present (level of pain varies)
☐Often present (pain free periods less than 6 hours)
☐Occasionally present (once to several times per day lasting up to an hour)
☐Rarely present (pain occurs every few days or weeks)

How would you describe your pain: ***(Check all that apply)***

☐Sharp, stabbing ☐Crampy ☐Heavy feeling in the pelvis ☐Dull, achy pain
☐Pulling, tugging pain ☐Throbbing pain ☐Burning pain ☐Falling out sensation
☐Other:

Does your pain ever wake you up from your sleep? ☐Yes ☐No

Does your pain ever radiate or spread to other regions of your body? ☐Yes ☐No

What makes your pain WORSE? ***(Check all that apply)***

☐Walking ☐Climbing stairs ☐Urination ☐Heavy lifting **☐Nothing makes it worse**
☐Full bladder ☐Stress ☐Housework ☐The weather ☐Getting in/out of the car
☐Exercise ☐Menstrual period ☐Contact with clothing ☐Intercourse/ Sexual contact
☐Bowel movements ☐Other:

What makes your pain BETTER? ***(Check all that apply)***

☐Lying down/rest ☐Emptying bladder ☐ Ice or Heating pad **☐Nothing makes it better**
☐Meditation ☐Laxatives/enema ☐It goes away by itself ☐When I feel supported
☐Hot bath ☐Massage ☐Bowel movements ☐When my stress is low
☐Exercise ☐Ibuprofen or Tylenol ☐Prescription pain medications
☐Being distracted, when I am busy doing other things ☐Other:

Last revised 6.19.2019 info@pelvicpain.org. www.pelvicpain.org

All information, content, and material on this form is for informational purposes only and is not intended to serve as a substitute for the consultation, diagnosis, and/or medical treatment of a qualified physician or healthcare professional.

5

 © 2023. Federação Brasileira de Ginecologia e Obstetrícia. All rights reserved.

INTERNATIONAL PELVICPAIN SOCIETY

PELVIC HEALTH HISTORY FORM

www.pelvicpain.org

11. Pain Location, Severity Scales and Past Treatments

Please mark ALL areas where you have pain on the Body Maps below as they apply to you. Please shade or circle each area of pain.

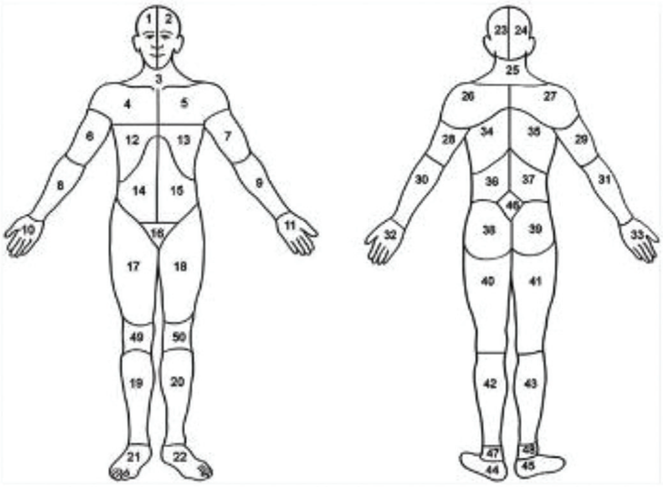

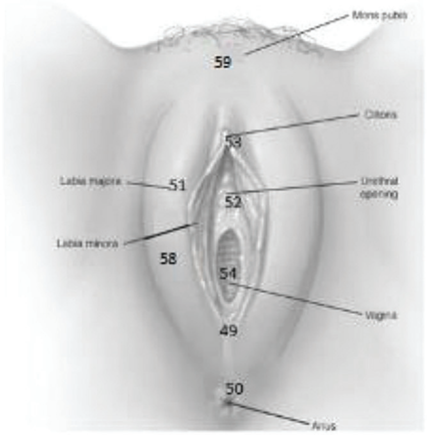

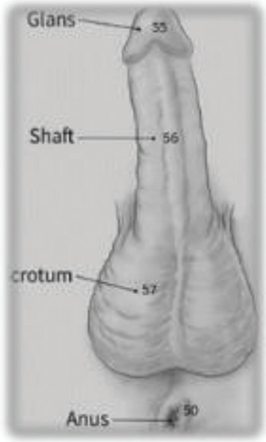

Last revised 6.19.2019 info@pelvicpain.org. www.pelvicpain.org

All information, content, and material on this form is for informational purposes only and is not intended to serve as a substitute for the consultation, diagnosis, and/or medical treatment of a qualified physician or healthcare professional.

6

 © 2023. Federação Brasileira de Ginecologia e Obstetrícia. All rights reserved.

INTERNATIONAL PELVIC PAIN SOCIETY

PELVIC HEALTH HISTORY FORM

www.pelvicpain.org

Short McGill Questionnaire

List each *pain location number from the body map in the first column*. Then, select the length, quality and severity of pain at each location. [IF YOU HAVE MORE THAN 3 AREAS OF PAIN, FILL THIS FOR YOUR 3 WORSE AREAS]

Example

(if 1 is by your pelvis it means the pain is in your pelvis)<br><br>1	☐1 year ☒1-3 years ☐4-7 years<br>☐8-10 years ☐More than 10 years	☒Throbbing ☐Shooting ☐Stabbing<br>☐Sharp ☐Cramping ☐Gnawing<br>☐Hot-Burning ☒Aching ☐Heavy<br>☐Tender ☐Splitting ☐Tiring-Exhausting<br>☐Sickening ☐Fearful ☐Punishing-Cruel	☐Mild<br>☐Moderate<br>☒Severe
This means you've had severe throbbing, aching, pelvic pain for 1-3 yeas.			
Location Number:	☐1 year ☐1-3 years ☐4-7 years<br>☐8-10 years ☐More than 10 years	☐Throbbing ☐Shooting ☐Stabbing<br>☐Sharp ☐Cramping ☐Gnawing<br>☐Hot-Burning ☐Aching ☐Heavy<br>☐Tender ☐Splitting ☐Tiring-Exhausting<br>☐Sickening ☐Fearful ☐Punishing-Cruel	☐Mild<br>☐Moderate<br>☐Severe
Location Number:	☐1 year ☐1-3 years ☐4-7 years<br>☐8-10 years ☐More than 10 years	☐Throbbing ☐Shooting ☐Stabbing<br>☐Sharp ☐Cramping ☐Gnawing<br>☐Hot-Burning ☐Aching ☐Heavy<br>☐Tender ☐Splitting ☐Tiring-Exhausting<br>☐Sickening ☐Fearful ☐Punishing-Cruel	☐Mild<br>☐Moderate<br>☐Severe
Location Number:	☐1 year ☐1-3 years ☐4-7 years<br>☐8-10 years ☐More than 10 years	Throbbing ☐Shooting ☐Stabbing<br>☐Sharp ☐Cramping ☐Gnawing<br>☐Hot-Burning ☐Aching ☐Heavy<br>☐Tender ☐Splitting ☐Tiring-Exhausting<br>☐Sickening ☐Fearful ☐Punishing-Cruel	☐Mild<br>☐Moderate<br>☐Severe

Indicate on this line by checking a box to describe how bad your MAIN pain is:

☐0 ☐1 ☐2 ☐3 ☐4 ☐5 ☐6 ☐7 ☐8 ☐9 ☐10

No Pain **Worse imaginable pain**

Last revised 6.19.2019 info@pelvicpain.org, www.pelvicpain.org

All information, content, and material on this form is for informational purposes only and is not intended to serve as a substitute for the consultation, diagnosis, and/or medical treatment of a qualified physician or healthcare professional.

7

 © 2023. Federação Brasileira de Ginecologia e Obstetrícia. All rights reserved.

INTERNATIONAL PELVIC PAIN SOCIETY

PELVIC HEALTH HISTORY FORM

www.pelvicpain.org

Pain Intensity Scale Short Form 3a

Rate the SEVERITY OF YOUR PAIN (*YOUR WORSE OR MAIN PAINFUL AREA*) on the scales below:

In the past ***7 days....***	Had no pain	Mild	Moderate	Severe	Very severe
**1. How intense was your pain at its worse?**	☐1	☐2	☐3	☐4	☐5
**2. How intense was your average pain?**	☐1	☐2	☐3	☐4	☐5
**3. What is your level of pain right now?**	☐1	☐2	☐3	☐4	☐5

Mark the one box that describes how much, during the past week, pain has interfered with:

	**0= does NOT interfere**										**completely interferes=10**
General activity	☐0	☐1	☐2	☐3	☐4	☐5	☐6	☐7	☐8	☐9	☐10
Mood	☐0	☐1	☐2	☐3	☐4	☐5	☐6	☐7	☐8	☐9	☐10
Walking activity	☐0	☐1	☐2	☐3	☐4	☐5	☐6	☐7	☐8	☐9	☐10
Normal activity (outside the home or with housework)	☐0	☐1	☐2	☐3	☐4	☐5	☐6	☐7	☐8	☐9	☐10
Relations with other people	☐0	☐1	☐2	☐3	☐4	☐5	☐6	☐7	☐8	☐9	☐10
Sleep	☐0	☐1	☐2	☐3	☐4	☐5	☐6	☐7	☐8	☐9	☐10
Enjoyment of life	☐0	☐1	☐2	☐3	☐4	☐5	☐6	☐7	☐8	☐9	☐10

Listed below are thirteen statements describing different thoughts and feelings that may be associated with pain. Please read each statement and circle a number 0,1,2,3, or 4 which indicates how much the statement applies to you when you are experiencing pain.

PCS

**When I am in pain...**	Not at all	To a slight degree	To a moderate degree	To a great degree	All the time
I worry all the time about whether the pain will end.	☐0	☐1	☐2	☐3	☐4
I feel I can't go on	☐0	☐1	☐2	☐3	☐4
It's terrible and I think it's never going to get any better	☐0	☐1	☐2	☐3	☐4
It's awful and I feel it overwhelms me	☐0	☐1	☐2	☐3	☐4
I feel I can't stand it anymore	☐0	☐1	☐2	☐3	☐4
I become afraid that the pain will get worse	☐0	☐1	☐2	☐3	☐4
I keep thinking of other painful events	☐0	☐1	☐2	☐3	☐4
I anxiously want the pain to go away	☐0	☐1	☐2	☐3	☐4
I can't seem to keep it out of my mind	☐0	☐1	☐2	☐3	☐4
I keep thinking about how much it hurts	☐0	☐1	☐2	☐3	☐4
I keep thinking about how badly I want the pain to stop	☐0	☐1	☐2	☐3	☐4
There's nothing I can do to reduce the intensity of the pain	☐0	☐1	☐2	☐3	☐4
I wonder whether something serious may happen	☐0	☐1	☐2	☐3	☐4

Last revised 6.19.2019 info@pelvicpain.org, www.pelvicpain.org

All information, content, and material on this form is for informational purposes only and is not intended to serve as a substitute for the consultation, diagnosis, and/or medical treatment of a qualified physician or healthcare professional.

8

 © 2023. Federação Brasileira de Ginecologia e Obstetrícia. All rights reserved.

INTERNATIONAL PELVIC PAIN SOCIETY

PELVIC HEALTH HISTORY FORM

www.pelvicpain.org

If assigned FEMALE at birth, complete this questionnaire to assess the impact of your pain on your sexuality.

**Interest in Sexual activity in the PAST 30 DAYS**						
**1. How interested have you been in sexual activity?**	Not at all ☐1	A little bit ☐2	Somewhat ☐3	Quite a bit ☐4	Very ☐5	
**2. How often have you felt like you wanted to have sex?**	Never ☐1	Rarely ☐2	Sometimes ☐3	Often ☐4	Always ☐5	
**Lubrication over the PAST 4 WEEKS...**						
**3. How often did you become lubricated 'wet' during sexual activity or intercourse?**	No sexual activity ☐0	Almost always or always ☐5	Most times (more than half the time) ☐4	Sometimes (about half the time) ☐3	A few times (less than half of the time) ☐2	Almost never or ever ☐1
**In the past 30 days...**						
**4. How difficult has it been for your vagina to be lubricated or 'wet' when you wanted it to?**	Not at all ☐1	A little bit ☐2	Somewhat ☐3	Quite a bit ☐4	Very ☐5	
**Vaginal Discomfort in the PAST 30 DAYS...**						
**5. How would you describe the comfort of your vagina during sexual activity?**	Have not had any sexual activity in the past 30 days ☐0	Never ☐1	Rarely ☐2	Sometimes ☐3	Often ☐4	Always ☐5
**6. How often have you had difficulty with sexual activity because of discomfort or pain in your vagina?**	Have not had any sexual activity in the past 30 days ☐0	Never ☐1	Rarely ☐2	Sometimes ☐3	Often ☐4	Always ☐5
**7. How often have you stopped sexual activity because of discomfort or pain in your vagina?**	Have not had any sexual activity in the past 30 days ☐0	Never ☐1	Rarely ☐2	Sometimes ☐3	Often ☐4	Always ☐5
**Orgasm in the PAST 30 DAYS...**						
**8. How would you rate your ability to have a satisfying orgasm/climax?**	Have not tried to have an orgasm/climax in the past 30 days ☐0	Excellent ☐5	Very good ☐4	Good ☐3	Fair ☐2	Poor ☐1
**Satisfaction in the PAST 30 DAYS...**						
**9. When you have had sexual activity how much have you enjoyed it?**	Have not had any sexual activity in the past 30 days ☐0	Not at all ☐1	A little bit ☐2	Somewhat ☐3	Quite a bit ☐4	Very ☐5
**10. When you have had sexual activity, how satisfying has it been?**	Have not had any sexual activity in the past 30 days □0	Not at all □1	A little bit □2	Somewhat □3	Quite a bit □4	Very □5

PROMIS Sexual Function Profile v1.0-Female

Last revised 6.19.2019 info@pelvicpain.org. www.pelvicpain.org

All information, content, and material on this form is for informational purposes only and is not intended to serve as a substitute for the consultation, diagnosis, and/or medical treatment of a qualified physician or healthcare professional.

9

 © 2023. Federação Brasileira de Ginecologia e Obstetrícia. All rights reserved.

INTERNATIONAL PELVICPAIN SOCIETY

PELVIC HEALTH HISTORY FORM

www.pelvicpain.org

If assigned MALE at birth, complete this questionnaire to assess the impact of your pain on your sexuality.

**Interest in Sexual activity in the PAST 30 DAYS**						
**How interested have you been in sexual activity?**	Not at all ☐1	A little bit ☐2	Somewhat ☐3	Quite a bit ☐4	Very ☐5	
**How often have you felt like you wanted to have sex?**	Never ☐1	Rarely ☐2	Sometimes ☐3	Often ☐4	Always ☐5	
**Erectile function, in the PAST 30 DAYS**						
**In the past 30 days...**						
**How difficult has it been for you to get an erection when you wanted to? (If you use pills, injections, or a penis pump to help you get an erection, please answer this question thinking about the times that you used these aids)**	Have not tried to get an erection in the past 30 days ☐0	Not at all ☐5	A little bit ☐4	Somewhat ☐3	Quite a bit ☐2	Very ☐1
**In the PAST 30 DAYS...**						
**How difficult has it been to keep an erection (stay hard) when you wanted to? (If you use pills, injections, or a penis pump to help you get an erection, please answer this question thinking about the times that you used these aids)**	Have not had erection in the past 30 days ☐0	Not at all ☐5	A little bit ☐4	Somewhat ☐3	Quite a bit ☐2	Very ☐1
**How would you rate the following in the LAST 4 WEEKS**						
**Your ability to have an erection**		Very poor ☐1	Poor ☐2	Fair ☐3	Good ☐4	Very good ☐5
**Orgasm in the PAST 30 DAYS...**						
**How would you rate your ability to have a satisfying orgasm/climax?**	Have not tried to have an orgasm/climax in the past 30 days ☐0	Excellent ☐5	Very good ☐4	Good ☐3	Fair ☐2	Poor ☐1
**Satisfaction in the PAST 30 DAYS...**						
**When you have had sexual activity how much have you enjoyed it?**	Have not had any sexual activity in the past 30 days ☐0	Not at all ☐1	A little bit ☐2	Somewhat ☐3	Quite a bit ☐4	Very ☐5
**When you have had sexual activity, how satisfying has it been?**	Have not had any sexual activity in the past 30 days ☐0	Not at all ☐1	A little bit ☐2	Somewhat ☐3	Quite a bit ☐4	Very ☐5

PROMIS Sexual Function Profile v1.0-Male

Last revised 6.19.2019 info@pelvicpain.org, www.pelvicpain.org

All information, content, and material on this form is for informational purposes only and is not intended to serve as a substitute for the consultation, diagnosis, and/or medical treatment of a qualified physician or healthcare professional.

10

 © 2023. Federação Brasileira de Ginecologia e Obstetrícia. All rights reserved.

INTERNATIONAL PELVICPAIN SOCIETY

PELVIC HEALTH HISTORY FORM

www.pelvicpain.org

REGARDLESS OF YOUR GENDER, please respond to each question or statement ABOUT YOUR GENERAL HEALTH by marking 1 box per row.

**In general, would you say your health is?**	Excellent □5	Very good □4	Good □3	Fair □2	Poor □1
**In general, would you say your quality of life is?**	Excellent □5	Very good □4	Good □3	Fair □2	Poor □1
**In general, how would you rate your physical health?**	Excellent □5	Very good □4	Good □3	Fair □2	Poor □1
**In general, how would you rate your mental health, including mood and your ability to think?**	Excellent □5	Very good □4	Good □3	Fair □2	Poor □1
**In general, how would you rate your satisfaction with your social activities and relationships?**	Excellent □5	Very good □4	Good □3	Fair □2	Poor □1
**In general, please rate how well you carry out your usual social activities and roles (this includes activities at home, at work and in your community, and responsibilities as a parent, child, spouse, employee, friend, etc.)**	Excellent □5	Very good □4	Good □3	Fair □2	Poor □1
**To what extend are you able to carry out your everyday physical activities such as walking, climbing stairs, carrying groceries, or moving a chair**	Completely □5	Mostly □4	Moderately □3	A little □2	Not at all □1
In the past 7 days...					
**How often have you been bothered by emotional problems such as feeling anxious, depressed or irritable?**	Never □1	Rarely □2	Sometimes □3	Often □4	Always □5
**How would you rate your fatigue on average?**	None □1	Mild □2	Moderate □3	Severe 4□	Very severe □5
**How would you rate your pain on average?**	□ 0-no pain	□ 1 □ 2 □ 3 □ 4 □ 5	□ 6 □ 7 □ 8	□ 9	□ 10 Worst imaginable pain

PROMIS Global Health v.1.1

[For health care providers-PROMIS scoring methods http://www.healthmeasures.net/score-and-interpret/calculate-scores]

Last revised 6.19.2019 info@pelvicpain.org, www.pelvicpain.org

All information, content, and material on this form is for informational purposes only and is not intended to serve as a substitute for the consultation, diagnosis, and/or medical treatment of a qualified physician or healthcare professional.

11

 © 2023. Federação Brasileira de Ginecologia e Obstetrícia. All rights reserved.

INTERNATIONAL PELVICPAIN SOCIETY

PELVIC HEALTH HISTORY FORM

www.pelvicpain.org

What medications have you tried in the PAST for your pelvic pain? ***(Check all that apply)***

Medication	Currently on Medication		Have tried this medication in the past		Did you find this medication helpful?		
Gabapentin (Neurontin®)	Yes☐	No☐	Yes☐	No☐	Yes☐	No☐	Somewhat☐
Pregabalin (Lyrica®)	Yes☐	No☐	Yes☐	No☐	Yes☐	No☐	Somewhat☐
Amitriptyline (Elavil®)	Yes☐	No☐	Yes☐	No☐	Yes☐	No☐	Somewhat☐
Duloxetine (Cymbalta®)	Yes☐	No☐	Yes☐	No☐	Yes☐	No☐	Somewhat☐
Milnacipran (Savella®)	Yes☐	No☐	Yes☐	No☐	Yes☐	No☐	Somewhat☐
Trazodone	Yes☐	No☐	Yes☐	No☐	Yes☐	No☐	Somewhat☐
Oral Muscle relaxer	Yes☐	No☐	Yes☐	No☐	Yes☐	No☐	Somewhat☐
Diazepam Suppository (Valium®)	Yes☐	No☐	Yes☐	No☐	Yes☐	No☐	Somewhat☐
Opioids	Yes☐	No☐	Yes☐	No☐	Yes☐	No☐	Somewhat☐
Other Medication not listed:							

What OTHER TREATMENTS have you tried for your pelvic pain IN THE PAST? ***(Check all that apply)***

☐Acupuncture ☐Massage ☐Nutrition/Diet ☐Physical Therapy ☐Biofeedback
☐Trigger Point Injections ☐TENS Unit ☐Botox Injections ☐Nerve Blocks
☐Epidural ☐Sex therapy ☐Joint Injections ☐Neurostimulation
☐Bladder instillations ☐Aqua therapy ☐Cognitive Behavioral Therapy
☐Radio Frequency Ablation (RFA) ☐NONE
☐Hormonal treatment-- if yes, what type of hormonal treatment? (***Check all that apply)***
☐Pills ☐Patch ☐Ring ☐ Injections ☐Estrogen ☐Progesterone
Other treatments:

12. Gastrointestinal History

Do you have any of the following GASTROINTESTINAL (BOWEL) symptoms? ***(Check all that apply)***

Nausea/vomiting? ☐Yes ☐No Constipation: ☐Yes ☐No
Diarrhea: ☐Yes ☐No Reflux / Heartburn: ☐Yes ☐No
Abdominal pain: ☐Yes ☐No
Bloating: ☐Yes ☐No

Do you have increased pain with bowel movements? ☐Yes ☐No

Do you have any rectal bleeding or blood in your stool? ☐Yes ☐No

Have you ever seen a gastroenterologist (GI specialist)? ☐Yes ☐No

Do you have pain or discomfort that is associated with any of the following?
Change in frequency of bowel movement? ☐Yes ☐No
Change in appearance of stool or bowel movement? ☐Yes ☐No

Does your pain improve or get worse around times of having a bowel movement? ☐Yes ☐No

Last revised 6.19.2019 info@pelvicpain.org. www.pelvicpain.org

All information, content, and material on this form is for informational purposes only and is not intended to serve as a substitute for the consultation, diagnosis, and/or medical treatment of a qualified physician or healthcare professional.

12

 © 2023. Federação Brasileira de Ginecologia e Obstetrícia. All rights reserved.

INTERNATIONAL PELVICPAIN SOCIETY

PELVIC HEALTH HISTORY FORM

www.pelvicpain.org

What do your stools look like MOST of the time? ***Select one type from the chart***

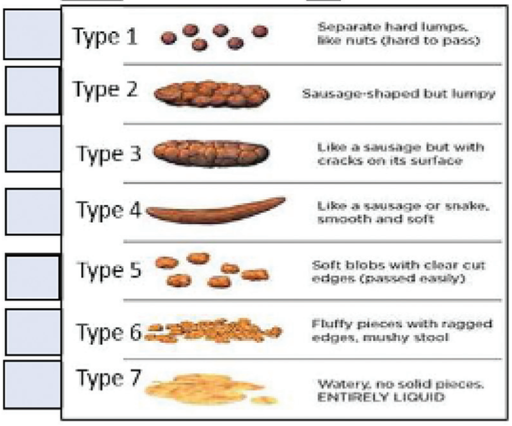

13. *Additional Symptoms and Diagnoses*

Do you have pain in your vulva/labia, clitoris, scrotum, penis or anus?	☐Yes	☐No
Do you have numbness in the same area?	☐Yes	☐No
Is your pain worsened by sitting?	☐Yes	☐No
Does the pain wake you up at night?	☐Yes	☐No
Have you ever had a pudendal nerve block?	☐Yes	☐No
If yes, did you have improvement in pain (even if temporary)?	☐Yes	☐No
Have you ever had any severe sport injuries (e.g. injuries during running, lifting, gymnastics)?	☐Yes	☐No
Have you ever had any motor vehicle accident injuries to your head, neck, spine or back?	☐Yes	☐No
Have you ever had any fall injuries (e.g. injuries to your back, tailbone, neck)?	☐Yes	☐No

Have you ever been diagnosed, or treated for any of these conditions? ***(Check all that apply)***

Condition		
Fibroids	☐Yes	☐No
Endometriosis	☐Yes	☐No
Fibromyalgia	☐Yes	☐No
Chronic fatigue syndrome / Myeloencephalitis	☐Yes	☐No
Interstitial cystitis / Bladder pain syndrome	☐Yes	☐No
Chronic low back pain	☐Yes	☐No
Chronic headaches or migraines	☐Yes	☐No
TMJ (Temporomandibular joint disorder)	☐Yes	☐No
Abnormal pap smear	☐Yes	☐No
Breast cancer	☐Yes	☐No
Other:		

Last revised 6.19.2019 info@pelvicpain.org, www.pelvicpain.org

All information, content, and material on this form is for informational purposes only and is not intended to serve as a substitute for the consultation, diagnosis, and/or medical treatment of a qualified physician or healthcare professional.

13

 © 2023. Federação Brasileira de Ginecologia e Obstetrícia. All rights reserved.

INTERNATIONAL PELVICPAIN SOCIETY

PELVIC HEALTH HISTORY FORM

www.pelvicpain.org

14. Urinary History

Do you experience any of the following URINARY SYMPTOMS? *(Check all that apply)*

Loss of urine when coughing, sneezing, or laughing?	☐Yes	☐No
Difficulty passing urine?	☐Yes	☐No
Frequent bladder infections?	☐Yes	☐No
Blood in the urine?	☐Yes	☐No
Still feeling full after urination?	☐Yes	☐No
Having to urinate again within minutes of urinating?	☐Yes	☐No
Urgency to go urinate	☐Yes	☐No

If assigned FEMALE at birth, complete the bladder function and symptom questionnaire. Please respond to questions 4-6 ONLY IF you engage in sexual intercourse.

Pelvic Pain / Urinary Frequency Questionnaire	0	1	2	3	4
1. How many times do you go to the bathroom **DURINGTHE DAY** (to void or empty your bladder)?	3-6 ☐	7-10 ☐	11-14 ☐	15-19 ☐	20 or more ☐
2. How many times do you go to the bathroom **AT NIGHT** (to void or empty your bladder)?	0 ☐	1 ☐	2 ☐	3 ☐	4 or more ☐
3. If you get up at night to void or empty your bladder does it bother you?	Never ☐	Mildly ☐	Moderately ☐	Severely ☐	
4. Are you sexually active? ☐Yes ☐ No					
5. If you are sexually active, do you now or have you ever, had pain or symptoms during or after sexual intercourse?	Never ☐	Occasionally ☐	Usually ☐	Always ☐	
6. If you have pain with intercourse, does it make you avoid sexual intercourse?	Never ☐	Occasionally ☐	Usually ☐	Always ☐	
7. Do you have pain associated with your bladder or in your pelvis (lower abdomen, labia, vagina, urethra, perineum)?	Never ☐	Occasionally ☐	Usually ☐	Always ☐	
8. Do you have urgency after voiding?	Never ☐	Occasionally ☐	Usually ☐	Always ☐	
9. If you have pain, is it usually	Never ☐	Mild ☐	Moderate ☐	Severe ☐	
10. Does your pain bother you?	Never ☐	Occasionally ☐	Usually ☐	Always ☐	
11. If you have urgency, is it usually		Mild ☐	Moderate ☐	Severe ☐	
12. Does your urgency bother you?	Never ☐	Occasionally ☐	Usually ☐	Always ☐	

Last revised 6.19.2019 info@pelvicpain.org. www.pelvicpain.org

All information, content, and material on this form is for informational purposes only and is not intended to serve as a substitute for the consultation, diagnosis, and/or medical treatment of a qualified physician or healthcare professional.

14

 © 2023. Federação Brasileira de Ginecologia e Obstetrícia. All rights reserved.

INTERNATIONAL PELVICPAIN SOCIETY

PELVIC HEALTH HISTORY FORM

www.pelvicpain.org

If assigned MALE at birth, please complete the Chronic Prostatitis Symptom Index (NIH):

**1.In the last week, have you experienced any pain or discomfort in the following areas?**	
a. Area between rectum and testicles (perineum)	☐1 Yes ☐2 No
b. Testicles	☐1 Yes ☐2 No
c. Tip of penis (not related to urination)	☐1 Yes ☐2 No
d. Below your waist, in your pubic or bladder area	☐1 Yes ☐2 No
**2.In the last week, have you experienced:**	
a. Pain or burning during urination?	☐1 Yes ☐2 No
b. Pain or discomfort during or after sexual climax (ejaculation)?	☐1 Yes ☐2 No
**3.How often have you had pain or discomfort in any of these areas (a-d) over the last week?**	☐0 Never<br>☐1 Rarely<br>☐2 Sometimes<br>☐3 Often<br>☐4 Usually<br>☐5 Always
**4.Which number best describes your AVERAGE pain or discomfort on the days that you had it, over the last week?**	**No Pain** **Worse imaginable pain**<br>☐0 ☐1 ☐2 ☐3 ☐4 ☐5 ☐6 ☐7 ☐8 ☐9 ☐10
**5.How often have you had the sensation of not emptying your bladder completely after you finished urinating, over the last week?**	☐0 Not at all<br>☐1 Less than 1 time in 5<br>☐2 Less than half the time<br>☐3 About half the time<br>☐4 More than Half the time<br>☐5 Almost always
**6.How often have you had to urinate again less than two hours after you finished urinating, over the last week**	☐0 Not at all<br>☐1 Less than 1 time in 5<br>☐2 Less than half the time<br>☐3 About half the time<br>☐4 More than Half the time<br>☐5 Almost always
**7.How much have your symptoms kept you from doing the kinds of things you would usually do, over the last week?**	☐0 None<br>☐1 Only a little<br>☐2 Some<br>☐3 A lot
**8.How much did you think about your symptoms over the last week?**	☐0 None<br>☐1 Only a little<br>☐2 Some<br>☐3 A lot
**8.If you were to spend the rest of your life with your symptoms just the way they have been during the last week, how would you feel about that?**	☐0 Delighted<br>☐1 Pleased<br>☐2 Mostly satisfied<br>☐3 Mixed (equally satisfied and dissatisfied<br>☐4 Mostly dissatisfied<br>☐5 Unhappy<br>☐6 Terrible
**Scoring**	
Pain: Total of items 1a, 1b, 1c, 1d, 2a, 2b, 3 and 4 =	
Urinary symptoms: Total of times 5 and 6 =	
Quality of life impact: Total of times 7, 8 and 9 =	

Last revised 6.19.2019 info@pelvicpain.org. www.pelvicpain.org

All information, content, and material on this form is for informational purposes only and is not intended to serve as a substitute for the consultation, diagnosis, and/or medical treatment of a qualified physician or healthcare professional.

15

 © 2023. Federação Brasileira de Ginecologia e Obstetrícia. All rights reserved.

INTERNATIONAL PELVICPAIN SOCIETY

PELVIC HEALTH HISTORY FORM

www.pelvicpain.org

1. Psychosocial History

What is the main source of stress in your life? ☐ Work ☐Family ☐Financial ☐Social ☐Relationships

Who are the people you talk to concerning your pain, during stressful times?
☐Spouse/ Partner ☐Relative ☐Support Group ☐Clergy ☐Doctor/Nurse
☐Friend ☐Mental Health Provider ☐I take care of myself

Have you ever experienced abuse or trauma as a child (13 years or younger)? (*Check all that apply*)
☐Emotional ☐Physical ☐Sexual ☐ Domestic Violence

Have you ever experienced abuse as an adult?
☐Emotional ☐Physical ☐Sexual ☐ Domestic Violence

Are you currently experiencing abuse?
☐Emotional ☐Physical ☐Sexual ☐ Domestic Violence

Have you ever received mental health treatment?
☐Medications ☐Therapy ☐Hospitalization

Are you currently still receiving mental health treatment? ☐Yes ☐No
If yes, please explain:

Do you have a history of?
☐Depression ☐ Anxiety ☐Panic Attacks ☐Bipolar Disorder
☐Trauma ☐PTSD ☐Disordered eating ☐None of these

Compared to other stressors in your life, how does your pain compare in importance?
☐ Most important ☐One of many problems

Are there relationships you think that may be contributing to your symptoms? ☐Yes ☐No

Do those that are in your daily life understand you? ☐Yes ☐No

If you have a partner, would you characterize them as supportive? ☐Yes ☐No

Does your partner notice if you are in pain? ☐Yes ☐No

How does your partner react when you hurt? Please explain:

Do you believe that your pain impacts other areas of your life?
☐Education ☐Family ☐Recreational activities
☐Work ☐Friends ☐Sexual intimacy

Last revised 6.19.2019 info@pelvicpain.org. www.pelvicpain.org

All information, content, and material on this form is for informational purposes only and is not intended to serve as a substitute for the consultation, diagnosis, and/or medical treatment of a qualified physician or healthcare professional.

16

 © 2023. Federação Brasileira de Ginecologia e Obstetrícia. All rights reserved.

INTERNATIONAL PELVIC PAIN SOCIETY

PELVIC HEALTH HISTORY FORM

www.pelvicpain.org

Please read each statement and circle a number 0, 1, 2, or 3 which indicates how much the statement applied to you over the past week. There are no wrong or right answers, do not spend too much time on any statement.

DASS-21	Not at all	Some of the time	A good part of the time	Most of the time
I found it hard to wind down	☐0	☐1	☐2	☐3
I was aware of dryness of my mouth	☐0	☐1	☐2	☐3
I couldn't seem to experience any positive feeling at all	☐0	☐1	☐2	☐3
I experienced breathing difficulty (e.g. excessively rapid breathing, breathlessness in the absence of physical exertion)	☐0	☐1	☐2	☐3
I found it difficult to work up the initiative to do things	☐0	☐1	☐2	☐3
I tended to overreact to situations	☐0	☐1	☐2	☐3
I experienced trembling (e.g. in the hands)	☐0	☐1	☐2	☐3
I felt that I was using a lot of nervous energy	☐0	☐1	☐2	☐3
I was worried about situations in which I might panic and make a fool of myself	☐0	☐1	☐2	☐3
I felt that I had nothing to look forward to	☐0	☐1	☐2	☐3
I found myself getting agitated	☐0	☐1	☐2	☐3
I found it difficult to relax	☐0	☐1	☐2	☐3
I felt down-hearted and blue	☐0	☐1	☐2	☐3
I was intolerant of anything that kept me from getting on with what I was doing	☐0	☐1	☐2	☐3
I felt I was close to panic	☐0	☐1	☐2	☐3
I was unable to become enthusiastic about anything	☐0	☐1	☐2	☐3
I felt I wasn't worth much as a person	☐0	☐1	☐2	☐3
I felt that I was rather touchy	☐0	☐1	☐2	☐3
I was aware of the action of my heart in the absence of physical exertion (e.g. a sense of heart rate increase, heart missing a beat)	☐0	☐1	☐2	☐3
I felt scared without good reason	☐0	☐1	☐2	☐3
I felt scared without good reason	☐0	☐1	☐2	☐3

Do you <u>CURRENTLY</u> use, or have you used any of the following substances in the <u>PAST 12 MONTHS</u>? *(Check all that apply)*

Substance			How many times a week?	Do you use this for pain control?
Do you drink any alcohol?	☐No	☐Yes	☐<1 ☐2-3 ☐>4	☐Yes ☐No
Tobacco or Nicotine Products	☐No	☐Yes	☐<1 ☐2-3 ☐>4	☐Yes ☐No
Cocaine / Crack	☐No	☐Yes	☐<1 ☐2-3 ☐>4	☐Yes ☐No
Heroin	☐No	☐Yes	☐<1 ☐2-3 ☐>4	☐Yes ☐No
Opioids	☐No	☐Yes	☐<1 ☐2-3 ☐>4	☐Yes ☐No
Methamphetamines	☐No	☐Yes	☐<1 ☐2-3 ☐>4	☐Yes ☐No
Stimulants	☐No	☐Yes	☐<1 ☐2-3 ☐>4	☐Yes ☐No
Ecstasy	☐No	☐Yes	☐<1 ☐2-3 ☐>4	☐Yes ☐No
Psychedelics	☐No	☐Yes	☐<1 ☐2-3 ☐>4	☐Yes ☐No
Marijuana/THC/Cannabis	☐No	☐Yes	☐<1 ☐2-3 ☐>4	☐Yes ☐No

Last revised 6.19.2019 info@pelvicpain.org, www.pelvicpain.org

All information, content, and material on this form is for informational purposes only and is not intended to serve as a substitute for the consultation, diagnosis, and/or medical treatment of a qualified physician or healthcare professional.

17

 © 2023. Federação Brasileira de Ginecologia e Obstetrícia. All rights reserved.

INTERNATIONAL PELVICPAIN SOCIETY

PELVIC HEALTH HISTORY FORM

www.pelvicpain.org

Thank you for taking the time to complete this form. This information will help your health care provider take better care of you.

For more information on chronic pelvic pain and how to prepare for your clinical evaluation, visit the 'patient resources' and 'pamphlets' section of the International Pelvic Pain Society web at www.pelvicpain.org.

FOR OFFICE USE ONLY:

Form reviewed by (Name):

Date of Review:

Health Care Provider Comments:

Last revised 6.19.2019 info@pelvicpain.org. www.pelvicpain.org

All information, content, and material on this form is for informational purposes only and is not intended to serve as a substitute for the consultation, diagnosis, and/or medical treatment of a qualified physician or healthcare professional.

18

 © 2023. Federação Brasileira de Ginecologia e Obstetrícia. All rights reserved.

Formulário de história de saúde pélvica

www.pelvicpain.org

Data de hoje: Número do prontuário (EXCLUSIVAMENTE PARA USO OFICIAL):

1. Informações de contato

Último sobrenome civil: **Primeiro nome civil:**

Data de nascimento: **Idade:**

E-mail: **Telefone:**

Como prefere ser chamado? (Selecionar <u>todas</u> as opções aplicáveis)

□ Ela □ Ele □ Eles/Elas □ Dr.

□ Último sobrenome civil □ Primeiro nome civil

□ Outro nome: □ Outro pronome de gênero:

Qual seu idioma de preferência para comunicação? (Selecionar <u>todas</u> as opções aplicáveis)

□ Português □ Inglês □ Espanhol □ Francês □ Outro:

2. Nome e informações de contato do profissional que encaminhou:

Nome: **Telefone:** **Endereço para contato:**

Quantos médicos ou profissionais de saúde você já consultou por motivo de <u>dor pélvica</u>?

□ Nenhum □ 1 □ 2 □ 3 □ 4 □ 5 □ 6 □7 □ 8 □ 9 □10 □ >10

3. Informações demográficas:

Qual a raça ou etnia que mais se aplica a você? (Selecionar <u>todas</u> as opções aplicáveis)

□ Branco □ Pardo □ Preto □ Amarelo □ Indígena □ Outro:

Qual é seu estado civil? (Selecionar <u>todas</u> as opções aplicáveis)

□ Solteiro(a) □ Casado(a) □ Separado(a) □ Divorciado(a) □ Viúvo(a) □ Relação estável

□ Relação sem compromisso □ Outro:

Descreva suas práticas sexuais: (Selecionar <u>todas</u> as opções aplicáveis)

□ NÃO sou sexualmente ativo(a) / abstinente

□ Assexual (desprovido de sentimentos ou associações sexuais)

□ Sexualmente ativo(a) com homens □ Sexualmente ativo(a) com mulheres

□ Sexualmente ativo com ambos

□ Outras:

Com quem você vive? (Selecionar <u>todas</u> as opções aplicáveis)

 © 2023. Federação Brasileira de Ginecologia e Obstetrícia. All rights reserved.

□ Sozinho(a) □ Companheiro(a) □ Pais □ Outro membro da família □ Amigos □ Morador de rua
□ Outro:

Qual o seu grau de instrução? (Selecione apenas um)
□ Menos de 12 anos de estudo □ Ensino médio completo □ Ensino superior completo □ Pós-graduação

Que tipo de trabalho você realiza? (Selecione somente um)
□ Desempregado(a) □ Trabalho fora de casa □ Do lar
□ Aposentado(a) □ Deficiente

4. História clínica

Anote seus problemas clínicos ou de saúde e especifique quando a doença foi diagnosticada, e se foi ou não controlada.

Problema clínico	Ano do diagnóstico	Foi controlada?
		□ Sim □ Não
		□ Sim □ Não
		□ Sim □ Não
		□ Sim □ Não
		□ Sim □ Não
		□ Sim □ Não

5. História cirúrgica

Informe se foi submetido(a) a qualquer das cirurgias abaixo

Procedimento		Data	Cirurgião	Achados
Cistoscopia (exame interno da bexiga)	□ Sim □ Não			
Laparoscopia com remoção de endometriose	□ Sim □ Não			
Histerectomia (retirada do útero e colo do útero)	□ Sim □ Não			
Seus ovários foram removidos?	□ Sim □ Não			
O colo do útero foi preservado (histerectomia supracervical)?	□ Sim □ Não			
Miomectomia	□ Sim □ Não			
Endoscopia	□ Sim			

 © 2023. Federação Brasileira de Ginecologia e Obstetrícia. All rights reserved.

	□ Não			
Colonoscopia	□ Sim<br>□ Não			
Remoção de cisto ovariano	□ Sim<br>□ Não			
Cesárea	□ Sim<br>□ Não			
Apendicectomia (retirada do apêndice)	□ Sim<br>□ Não			
Prostatectomia	□ Sim<br>□ Não			
Colectomia (remoção do cólon)	□ Sim<br>□ Não			
Vasectomia	□ Sim<br>□ Não			
Outra:				

6. Histórico de menstruação, controle de natalidade, e infecções sexualmente transmissíveis

Caso você <u>NÃO</u> menstrue, selecione o(s) motivo(s): (Selecionar <u>todas</u> as opções aplicáveis)

□ Fiz histerectomia □ Menopausa □ Se atribuído sexo MASCULINO ao nascimento, pular para **

□ Supressão menstrual contínua por método contraceptivo (por exemplo: Depo-Provera, pílula, DIU à base de progesterona)

□ Submetida à ablação endometrial

Qual a data da sua última menstruação?

Qual a sua idade quando seus ciclos menstruais começaram?

Caso você menstrue, <u>ATUALMENTE</u> você apresenta quaisquer dos sintomas abaixo <u>DURANTE</u> a menstruação? (Selecionar <u>todas</u> as opções aplicáveis)

□ Sangramento profuso □ Dor intensa □ Sangramento irregular (mais de uma vez por mês)

□ Sangramento > 7 dias

□ Alterações de humor □ Fadiga □ Dor na mama □ Constipação □ Diarreia □ Dores de cabeça

Caso tenha dores menstruais, há quanto tempo sente esse tipo de dor? Especifique anos ou meses.

<u>ATUALMENTE</u>, você perde aula ou falta ao trabalho regularmente (mais de 3 vezes por mês) devido a cólicas menstruais?

□ Sim □ Não

 © 2023. Federação Brasileira de Ginecologia e Obstetrícia. All rights reserved.

Caso tenha cólicas menstruais, já tentou alguma das alternativas abaixo para aliviar a dor durante a menstruação? (Selecionar todas as opções aplicáveis)

□ Pílula anticoncepcional □ Anel vaginal □ Injeção trimestral □ DIU hormonal

□ AINES (anti-inflamatórios não esteroides, como ibuprofeno, naproxeno)

□ Analgésicos comuns (Paracetamol, escopolamina) □ Outro:

**** Qual método anticoncepcional / contraceptivo você usa? (Selecionar todas as opções aplicáveis):**

□ Nenhum □ Vasectomia □ Preservativo

□ Pílula anticoncepcional □ Injeção trimestral

□ Implante (Implanon) □ Anel vaginal (NuvaRing) □ Laqueadura tubária

□ DIU hormonal □ DIU não hormonal □ Outro:

Você já teve infecções sexualmente transmissíveis (ISTs)? (Selecionar todas as opções aplicáveis)

□ Clamídia □ Gonorreia □ Herpes □ HPV (Papilomavírus humano) □ Sífilis

□ DIP (doença inflamatória pélvica) □ HIV □ Hepatite B □ Hepatite C

7. Alergias e medicações atuais

Liste suas alergias:

Alergia	Reação, o que acontece quando você tem essa alergia?	Já foi tratado para essa alergia?

Liste todas os medicamentos que toma ATUALMENTE, incluindo fitoterápicos:

Medicamento ou fitoterápico	Dose	Para qual doença

 © 2023. Federação Brasileira de Ginecologia e Obstetrícia. All rights reserved.

8. História obstétrica

Quantas gestações você teve? □ 0 □ 1 □ 2 □3 □ 4 □ 5 □ 6 ou mais

Quantos partos você teve? □ 0 □ 1 □ 2 □3 □ 4 □ 5 □ 6 ou mais

Quantos partos foram vaginais? □ 0 □ 1 □ 2 □3 □ 4 □ 5 □ 6 ou mais

Quantos partos foram cesáreas? □ 0 □ 1 □ 2 □3 □ 4 □ 5 □ 6 ou mais

Quantos abortos? □ 0 □ 1 □ 2 □3 □ 4 □ 5 □ 6 ou mais

Teve complicações durante a gestação, trabalho de parto, parto, ou após o parto?

□ Laceração 3°- 4 □ Vácuo extrator/ Fórceps □ Complicações com a ferida □ Outras

9. Histórico familiar

Alguém de sua família já teve alguma das doenças abaixo? (Selecionar <u>todas</u> as opções aplicáveis)

□ Endometriose □ Fibromialgia □ Dor pélvica crônica □ Síndrome do intestino irritável

□ Cistite intersticial □ Câncer de colo □ Câncer de mama □ Câncer de útero □ Câncer de ovário

□ Depressão □ Síndrome da fadiga crônica □ Ansiedade/crises de pânico

□ Distúrbio da articulação temporomandibular (ATM) □ Enxaqueca

□ Transtorno do estresse pós-traumático (TEPT)

□ Outra doença crônica:

10. História, descrição e fatores contribuintes de dor

Quando a sua dor começou? Mês: Ano: □ Não tenho certeza

Descreva a dor com suas próprias palavras:

Como sua dor principal começou, você se lembra de algum incidente específico ocorrido na época em que a dor começou? (Selecione <u>um</u>)

□ Lesão em casa □ Lesão no trabalho/escola □ Lesão em outro local □ Acidente automobilístico

□ Após cirurgia □ Câncer □ Doença diferente de câncer

□ Sem causa aparente/ não me recordo de incidente específico □ Outro:

Como sua dor começou? (Selecione somente um) □ De forma súbita/rápida?

□ De forma gradual/lenta?

Há quanto tempo sofre esta dor principal? (Selecione somente <u>uma</u> alternativa)

□ Menos de 3 meses □ 3 a 12 meses □ 12 meses a 2 anos □ 2 a 5 anos □ Mais de 5 anos

Desde que começou, sua dor: (Selecione somente <u>uma</u> alternativa)

□ Permanece inalterada □ Está melhorando □ Está piorando □ Não sei

 © 2023. Federação Brasileira de Ginecologia e Obstetrícia. All rights reserved.

Qual frase melhor descreve sua dor? (Selecione somente uma)

□ Sempre presente (sempre com a mesma intensidade)
□ Sempre presente (intensidade da dor varia)
□ Geralmente presente (períodos de menos de 6 horas sem dor)
□ Eventualmente presente (uma a várias vezes por dia, com duração de até uma hora)
□ Raramente presente (dor a cada poucos dias ou semanas)

Como descreveria sua dor: (Selecionar todas as opções aplicáveis)

□ Aguda, em pontada □ Cólica □ Sensação de peso na pelve □ Surda, persistente
□ Em fisgada □ Pulsátil □ Em queimação □ Sensação de algo caindo
□ Outra:

Você chega a acordar por causa da dor? □ Sim □ Não

Sua dor chega a irradiar ou se espalhar para outras regiões do seu corpo? □ Sim □ Não

O que PIORA sua dor? (Selecionar todas as opções aplicáveis)

□ Caminhar □ Subir escadas □ Urinar □ Levantar muito peso □ **Nada piora minha dor**
□ Bexiga cheia □ Estresse □ Tarefa doméstica □ O clima □ Entrar/sair do carro
□ Exercícios □ Período menstrual □ Contato com a roupa □ Relação/contato sexual
□ Evacuação □ Outro:

O que MELHORA a sua dor? (Selecionar todas as opções aplicáveis)

□ Deitar/descansar □ Esvaziar a bexiga □ Bolsa de gelo ou água quente □ **Nada melhora minha dor**
□ Meditação □Laxantes/enema □ Desaparece sozinha □ Quando me sinto apoiado
□ Banho quente □ Massagem □ Evacuação □ Quando meu nível de estresse é baixo
□ Exercícios □Ibuprofeno ou Tylenol □ Analgésicos
□ Me distrair, quando estou ocupado(a) com outras coisas □ Outro:

11. Localização da dor, Escalas de intensidade e Tratamentos realizados

Marque TODAS as áreas onde você sente dor nos Mapas Corporais abaixo. Preencha ou faça um círculo em cada área de dor

 © 2023. Federação Brasileira de Ginecologia e Obstetrícia. All rights reserved.

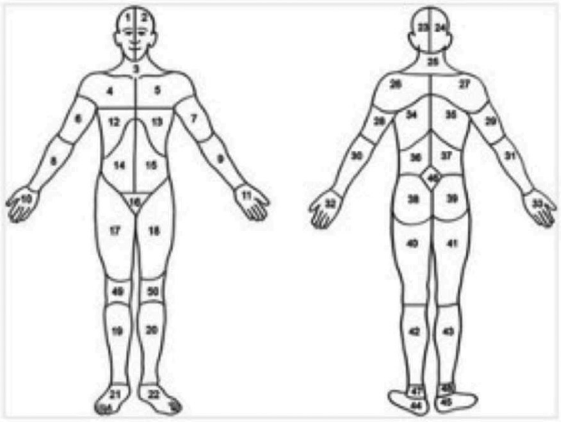

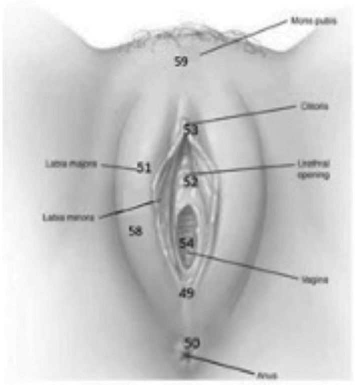

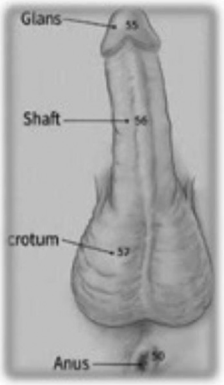

Tradução das figuras:
Genitália feminina: 49 – Períneo ; 50 – Ânus; 51 – Grande lábio; 52 – Orifício uretral; 53 – Clitóris; 54 – Vagina; 58 – Pequeno lábio; 59 – Monte púbico.
Genitália masculina: 50 – Ânus; 55 – Glande; 56 – Haste peniana; 57 – Escroto.

Questionário McGill - versão curta

<table>
<tr><td colspan="4">Anote <u>o número correspondente à localização de cada dor, no mapa corporal, na primeira coluna</u>. Em seguida, selecione a duração, a qualidade e a intensidade da dor em cada localização [CASO VOCÊ TENHA DOR EM MAIS DE 3 ÁREAS, PREENCHA DE ACORDO COM AS 3 PIORES ÁREAS]</td></tr>
<tr><td colspan="4">EXEMPLO</td></tr>
<tr><td>(se 1 for na região da pelve, isso significa que a dor está localizada na sua pelve)

1</td><td>□ 1 ano
⊠1-3 anos
□ 4-7 anos
□ 8-10 anos
□ Mais de 10 anos</td><td>⊠ Pulsátil □ Que irradia
□ Em pontada □ Aguda □ Cólica
□ Agoniante □ Em queimação
⊠ Persistente □ Pesada
□ Sensível □ Lancinante □ Exaustiva
□ Nauseante □ Terrível
□ Castigo - Cruel</td><td>□ Leve
□ Moderada
⊠ Intensa</td></tr>
<tr><td colspan="4">Isso significa que você teve dor pélvica pulsátil, persistente e intensa por 1 a 3 anos.</td></tr>
</table>

 © 2023. Federação Brasileira de Ginecologia e Obstetrícia. All rights reserved.

Número correspondente à localização	□ 1 ano<br>□1-3 anos<br>□ 4-7 anos<br>□ 8-10 anos<br>□ Mais de 10 anos	□ Pulsátil □ Que irradia<br>□ Em pontada □ Aguda □ Cólica<br>□ Agoniante □ Em queimação<br>□ Persistente □ Pesada<br>□ Sensível □ Lancinante □ Exaustiva<br>□ Nauseante □ Terrível<br>□ Castigo - Cruel	□ Leve<br>□ Moderada<br>□ Intensa
Número correspondente à localização	□ 1 ano<br>□1-3 anos<br>□ 4-7 anos<br>□ 8-10 anos<br>□ Mais de 10 anos	□ Pulsátil □ Que irradia<br>□ Em pontada □ Aguda □ Cólica<br>□ Agoniante □ Em queimação<br>□ Persistente □ Pesada<br>□ Sensível □ Lancinante □ Exaustiva<br>□ Nauseante □ Terrível<br>□ Castigo - Cruel	□ Leve<br>□ Moderada<br>□ Intensa
Número correspondente à localização	□ 1 ano<br>□1-3 anos<br>□ 4-7 anos<br>□ 8-10 anos<br>□ Mais de 10 anos	□ Pulsátil □ Que irradia<br>□ Em pontada □ Aguda □ Cólica<br>□ Agoniante □ Em queimação<br>□ Persistente □ Pesada<br>□ Sensível □ Lancinante □ Exaustiva<br>□ Nauseante □ Terrível<br>□ Castigo - Cruel	□ Leve<br>□ Moderada<br>□ Intensa

Indique nesta linha, selecionado uma das caixas, a descrição da intensidade de sua dor PRINCIPAL:

□ 0 □ 1 □ 2 □ 3 □ 4 □ 5 □ 6 □ 7 □ 8 □ 9 □ 10

Sem dor Pior dor possível

Classifique a INTENSIDADE DA SUA DOR (SUA PIOR OU PRINCIPAL ÁREA DOLOROSA) nas escalas abaixo:

Escala de Intensidade de Dor - versão curta 3a

Nos últimos 7 dias....

	Não tive dor	Leve	Moderada	Intensa	Muito intensa
1. Qual a intensidade de sua dor no pior momento?	□ 1	□ 2	□ 3	□ 4	□ 5
2. Qual a intensidade de sua dor, em média?	□ 1	□ 2	□ 3	□ 4	□ 5
3. Qual a intensidade de sua dor neste momento?	□ 1	□ 2	□ 3	□ 4	□ 5

 © 2023. Federação Brasileira de Ginecologia e Obstetrícia. All rights reserved.

Selecione uma caixa que descreva o quanto, durante a semana passada, a dor interferiu:

	0 = NÃO interferiu				Interferiu completamente = 10						
Atividade geral	□ 0	□ 1	□ 2	□ 3	□ 4	□ 5	□ 6	□ 7	□ 8	□ 9	□ 10
Humor	□ 0	□ 1	□ 2	□ 3	□ 4	□ 5	□ 6	□ 7	□ 8	□ 9	□ 10
Caminhada	□ 0	□ 1	□ 2	□ 3	□ 4	□ 5	□ 6	□ 7	□ 8	□ 9	□ 10
Atividade normal (fora de casa ou trabalho doméstico)	□ 0	□ 1	□ 2	□ 3	□ 4	□ 5	□ 6	□ 7	□ 8	□ 9	□ 10
Relações com outras pessoas	□ 0	□ 1	□ 2	□ 3	□ 4	□ 5	□ 6	□ 7	□ 8	□ 9	□ 10
Sono	□ 0	□ 1	□ 2	□ 3	□ 4	□ 5	□ 6	□ 7	□ 8	□ 9	□ 10
Prazer de viver	□ 0	□ 1	□ 2	□ 3	□ 4	□ 5	□ 6	□ 7	□ 8	□ 9	□ 10

Abaixo encontram-se treze frases que descrevem diferentes pensamentos e sentimentos que podem estar associados à dor. Leia cada frase e circule um número 0, 1, 2, 3, ou 4, que indique o quanto essa frase se aplica a você, quando está com dor.

PCS

**Quando sinto dor…**	**Nunca**	**Um pouco**	**Moderada-mente**	**Muito**	**O tempo todo**
Me preocupo o tempo todo se a dor vai passar	□ 0	□ 1	□ 2	□ 3	□ 4
Sinto que não é possível continuar	□ 0	□ 1	□ 2	□ 3	□ 4
É terrível, e penso que nunca vai melhorar	□ 0	□ 1	□ 2	□ 3	□ 4
É péssimo, e sinto que me derrota	□ 0	□ 1	□ 2	□ 3	□ 4
Sinto que não dá mais para aguentar	□ 0	□ 1	□ 2	□ 3	□ 4
Fico com medo que a dor piore	□ 0	□ 1	□ 2	□ 3	□ 4
Fico pensando em outros eventos dolorosos	□ 0	□ 1	□ 2	□ 3	□ 4

 © 2023. Federação Brasileira de Ginecologia e Obstetrícia. All rights reserved.

Desejo ansiosamente que a dor passe	□ 0	□ 1	□ 2	□ 3	□ 4
Não consigo pensar em outra coisa	□ 0	□ 1	□ 2	□ 3	□ 4
Fico pensando no quanto dói	□ 0	□ 1	□ 2	□ 3	□ 4
Fico pensando no quanto desejo que a dor passe	□ 0	□ 1	□ 2	□ 3	□ 4
Não há nada que eu possa fazer para diminuir a intensidade da dor	□ 0	□ 1	□ 2	□ 3	□ 4
Penso se algo grave pode acontecer	□ 0	□ 1	□ 2	□ 3	□ 4

PROMIS Perfil da Função Sexual v1.0 - Mulher

Se atribuído sexo FEMININO ao nascimento, complete este questionário para avaliar o impacto da dor sobre sua sexualidade.

**Interesse em atividade sexual nos ÚLTIMOS 30 DIAS**						
1. Qual o seu grau de interesse por atividades sexuais?	Nenhum □ 1	Um pouco □ 2	Médio □ 3	Bastante □ 4	Muito □5	
2. Com que frequência você sentiu vontade de fazer sexo	Nunca □ 1	Raramente □ 2	Às vezes □ 3	Com frequência □ 4	Sempre □ 5	
**Lubrificação nas ÚLTIMAS 4 SEMANAS…**						
3. Com que frequência você ficou lubrificada ou "molhada" durante a atividade ou relação sexual?	Não tive atividade sexual □ 0	Quase sempre ou sempre □ 5	Na maioria das vezes (mais da metade das vezes) □ 4	Às vezes (cerca de metade das vezes) □ 3	Algumas vezes (menos da metade das vezes) □ 2	Quase nunca ou nunca □ 1
**Nos últimos 30 dias…**						

 © 2023. Federação Brasileira de Ginecologia e Obstetrícia. All rights reserved.

4. Qual o grau de dificuldade para fazer com que sua vagina ficasse lubrificada ou "molhada" no momento desejado?	Nenhum ◻ 1	Um pouco ◻ 2	Médio ◻ 3	Bastante ◻ 4	Muito ◻ 5	
**<u>Desconforto vaginal</u> nos ÚLTIMOS 30 DIAS…**						
5. Como descreveria o seu conforto vaginal durante a atividade sexual?	Não tive atividade sexual nos últimos 30 dias ◻ 0	Nunca ◻ 1	Raramente ◻ 2	Às vezes ◻ 3	Com frequência ◻ 4	Sempre ◻ 5
6. Com que frequência teve dificuldade na atividade sexual por desconforto ou dor na vagina?	Não tive atividade sexual nos últimos 30 dias ◻ 0	Nunca ◻ 1	Raramente ◻ 2	Às vezes ◻ 3	Com frequência ◻ 4	Sempre ◻ 5
7. Com que frequência interrompeu a atividade sexual por desconforto ou dor na vagina?	Não tive atividade sexual nos últimos 30 dias ◻ 0	Nunca ◻ 1	Raramente ◻ 2	Às vezes ◻ 3	Com frequência ◻ 4	Sempre ◻ 5
**<u>Orgasmo</u> nos ÚLTIMOS 30 DIAS...**						
8. Como avaliaria sua capacidade de atingir um orgasmo/clímax satisfatório?	Não tentei atingir um orgasmo/clímax nos últimos 30 dias	Excelente ◻ 5	Muito boa ◻ 4	Boa ◻ 3	Razoável ◻ 2	Ruim ◻ 1
**<u>Satisfação</u> nos ÚLTIMOS 30 DIAS…**						
9. Nas ocasiões em que teve atividade sexual, qual o seu grau de prazer?	Não tive atividade sexual nos últimos 30 dias ◻ 0	Nenhum ◻ 1	Um pouco ◻ 2	Médio ◻ 3	Bastante ◻ 4	Muito ◻ 5
10. Nas ocasiões em que teve atividade sexual, qual seu grau de satisfação?	Não tive atividade sexual nos últimos 30 dias ◻ 0	Nenhum ◻ 1	Um pouco ◻ 2	Médio ◻ 3	Bastante ◻ 4	Muito ◻ 5

PROMIS Perfil da Função Sexual v1.0 - Homem

 © 2023. Federação Brasileira de Ginecologia e Obstetrícia. All rights reserved.

	□ 0					
**Satisfação** nos ÚLTIMOS 30 DIAS...						
Nas ocasiões em que teve atividade sexual, qual o seu grau de prazer?	Não tive atividade sexual nos últimos 30 dias □ 0	Nenhum □ 1	Um pouco □ 2	Médio □ 3	Bastante □ 4	Muito □ 5
Nas ocasiões em que você teve, atividade sexual, qual o seu grau de satisfação?	Não tive atividade sexual nos últimos 30 dias □ 0	Nenhum □ 1	Um pouco □ 2	Médio □ 3	Bastante □ 4	Muito □ 5

PROMIS Saúde Global v.1.1

INDEPENDENTE DE SEU GÊNERO, responda a cada pergunta ou frase SOBRE SUA SAÚDE GERAL selecionando 1 caixa por linha.

De forma geral, como diria que sua saúde é?	Excelente □ 5	Muito boa □4	Boa □ 3	Razoável □ 2	Ruim □ 1
De forma geral, como diria que sua qualidade de vida é?	Excelente □ 5	Muito boa □4	Boa □ 3	Razoável □ 2	Ruim □ 1
De forma geral, como diria que sua saúde física é?	Excelente □ 5	Muito boa □4	Boa □ 3	Razoável □ 2	Ruim □ 1
De forma geral, como avaliaria a sua saúde mental, incluindo humor e sua capacidade de pensar?	Excelente □ 5	Muito boa □4	Boa □ 3	Razoável □ 2	Ruim □ 1
De forma geral, como avaliaria sua satisfação com suas atividades sociais e relacionamentos?	Excelente □ 5	Muito boa □4	Boa □ 3	Razoável □ 2	Ruim □ 1
De forma geral, avalie como desempenha suas atividades e papeis sociais (isso inclui atividades realizadas em casa, no trabalho e em sua comunidade, além das responsabilidades parentais, de	Excelente □ 5	Muito boa □4	Boa □ 3	Razoável □ 2	Ruim □ 1

 © 2023. Federação Brasileira de Ginecologia e Obstetrícia. All rights reserved.

filho(a), esposo(a), funcionário(a), amigo(a), etc.)					
Até que ponto é capaz de realizar suas atividades físicas rotineiras, como caminhar, subir escadas, carregar as compras, ou mover uma cadeira	Completamente □ 5	Na maioria das vezes □4	Moderadamente □ 3	Um pouco □ 2	De jeito nenhum □ 1
**Nos últimos 7 dias...**					
Com que frequência foi incomodado por problemas emocionais, sentindo ansiedade, depressão ou irritabilidade?	Nunca □ 1	Raramente □ 2	Às vezes □ 3	Com frequência □4	Sempre □ 5

Como avaliaria seu grau de dor, em média?

□ 0 □ 1 □ 2 □ 3 □ 4 □ 5 □ 6 □ 7 □ 8 □ 9 □ 10

Sem dor Pior dor possível

[Para profissionais da saúde - Métodos de pontuação PROMIS - http://www.healthmeasures.net/score-and-interpret/calculate-scores]

Quais medicamentos já tomou no PASSADO para sua dor pélvica? (Selecionar todas as opções aplicáveis)

Medicamento	Tomando atualmente	Tomei no passado	Essa medicação foi útil?
Gabapentina (Neurotin®)	□ Sim □ Não	□ Sim □ Não	□ Sim □ Não □ Um pouco
Pregabalina (Lyrica®)	□ Sim □ Não	□ Sim □ Não	□ Sim □ Não □ Um pouco
Amitriptilina (Elavil®)	□ Sim □ Não	□ Sim □ Não	□ Sim □ Não □ Um pouco
Duloxetina (Cymbalta®)	□ Sim □ Não	□ Sim □ Não	□ Sim □ Não □ Um pouco
Milnaciprano (Savella®)	□ Sim □ Não	□ Sim □ Não	□ Sim □ Não □ Um pouco
Trazodona	□ Sim □ Não	□ Sim □ Não	□ Sim □ Não □ Um pouco
Relaxante muscular oral	□ Sim □ Não	□ Sim □ Não	□ Sim □ Não □ Um pouco
Diazepam supositório (Valium®)	□ Sim □ Não	□ Sim □ Não	□ Sim □ Não □ Um pouco
Opioides	□ Sim □ Não	□ Sim □ Não	□ Sim □ Não □ Um pouco
Outro medicamento não incluído na lista:			

Quais OUTROS TRATAMENTOS você experimentou para sua dor pélvica NO PASSADO?

 © 2023. Federação Brasileira de Ginecologia e Obstetrícia. All rights reserved.

(Selecionar todas as opções aplicáveis)

□ Acupuntura □Massagem □ Nutrição/Dieta □ Fisioterapia □ *Biofeedback*
□ Injeções em pontos gatilho □TENS □ Injeções de botox
□ Bloqueios perineurais
□ Epidural □ Terapia sexual □ Injeções intra-articulares
□ Neuroestimulação
□ Instilações intravesicais □Hidroterapia □ Terapia cognitiva comportamental
□ Ablação por radiofrequência (RFA) □ NENHUM
□ Tratamento hormonal - em caso afirmativo, que tipo de tratamento hormonal? (Selecionar todas as opções aplicáveis)
□ Pílulas □ Adesivo □Anel □ Injeções □Estrógeno
□ Progesterona
Outros tratamentos:

12. História gastrointestinal

Sente algum dos seguintes sintomas GASTROINTESTINAIS? (Selecionar todas as opções aplicáveis)

Náusea/vômitos? □ Sim □ Não
Constipação: □ Sim □ Não
Diarreia: □ Sim □ Não
Dor abdominal: □ Sim □ Não
Distensão abdominal: □ Sim □ Não
Constipação: □ Sim □ Não
Refluxo / Azia: □ Sim □ Não

Sua dor aumenta com evacuação? □ Sim □ Não

Tem sangramento retal ou sangue nas fezes? □ Sim □ Não

Já foi a um gastroenterologista? (especialista do trato gastrointestinal) □ Sim □ Não

Sente dor ou desconforto associado a algum dos itens abaixo?
Alteração da frequência das evacuações? □ Sim □ Não
Alteração da aparência das fezes ou evacuações? □ Sim □ Não

Sua dor melhora ou piora perto do momento de evacuar? □ Sim □ Não

 © 2023. Federação Brasileira de Ginecologia e Obstetrícia. All rights reserved.

Qual o aspecto das suas fezes na MAIORIA das vezes? Selecione um tipo do quadro.

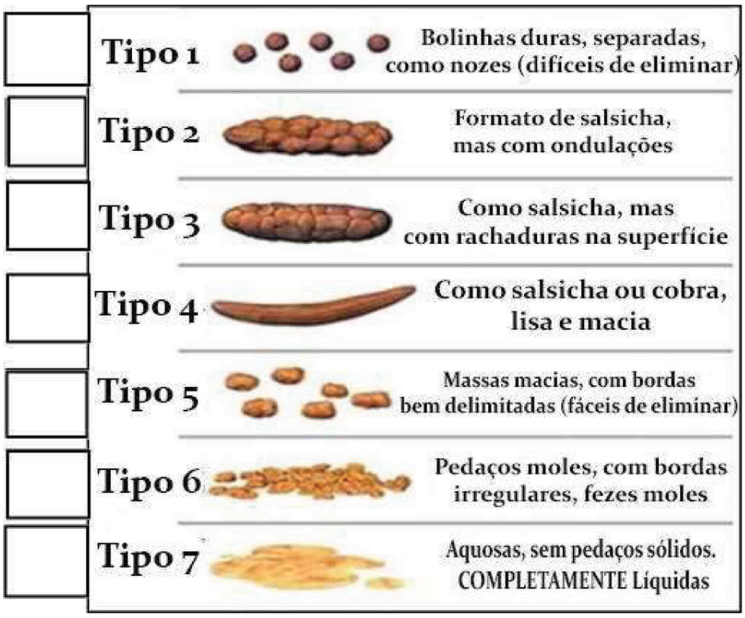

13. Outros sintomas e diagnósticos

Sente dor na vulva/lábios, clítoris, escroto, pênis ou ânus?	□ Sim	□ Não
Sente formigamento na mesma área?	□ Sim	□ Não
Sua dor piora ao se sentar?	□ Sim	□ Não
Sua dor o desperta durante a noite?	□ Sim	□ Não
Já foi submetido a bloqueio do nervo pudendo?	□ Sim	□ Não
Em caso afirmativo, sua dor melhorou (mesmo que temporariamente)?	□ Sim	□ Não
Já sofreu alguma lesão grave relacionada ao esporte (ex: lesões durante a corrida, musculação, ginástica)?	□ Sim	□ Não
Já sofreu alguma lesão craniana, cervical, na coluna ou nas costas por acidente automobilístico?	□ Sim	□ Não

 © 2023. Federação Brasileira de Ginecologia e Obstetrícia. All rights reserved.

Já sofreu alguma lesão por queda (ex: lesão cervical, nas costas ou no cóccix)?	□ Sim	□ Não

Já foi diagnosticado, ou tratado para alguma das doenças abaixo? (Selecionar todas as opções aplicáveis)

**Doença**		
Mioma	□ Sim	□ Não
Endometriose	□ Sim	□ Não
Fibromialgia	□ Sim	□ Não
Síndrome da fadiga crônica / Mieloencefalite	□ Sim	□ Não
Cistite intersticial / Síndrome da dor vesical	□ Sim	□ Não
Lombalgia crônica	□ Sim	□ Não
Dor de cabeça crônica ou enxaqueca	□ Sim	□ Não
Distúrbio da articulação temporomandibular (ATM)	□ Sim	□ Não
Resultado anormal no exame de Papanicolau	□ Sim	□ Não
Câncer de mama	□ Sim	□ Não
Outro:		

14. História urinária

Sente algum dos SINTOMAS URINÁRIOS abaixo? (Selecionar todas as opções aplicáveis)

Escape de urina ao tossir, espirrar ou rir?	□ Sim	□ Não
Dificuldade de urinar?	□ Sim	□ Não
Infecções urinárias frequentes?	□ Sim	□ Não
Sangue na urina?	□ Sim	□ Não
Sensação de bexiga cheia depois de urinar?	□ Sim	□ Não
Necessidade de urinar novamente minutos depois de ter urinado?	□ Sim	□ Não
Urgência para urinar?	□ Sim	□ Não

Se atribuído sexo FEMININO ao nascimento, complete o questionário de função e sintomas vesicais. Responda às perguntas 4-6 SOMENTE SE estiver mantendo relações sexuais.

**Questionário de dor pélvica / frequência urinária**	**0**	**1**	**2**	**3**	**4**

 © 2023. Federação Brasileira de Ginecologia e Obstetrícia. All rights reserved.

1. Quantas vezes vai ao banheiro **DURANTE O DIA** (para urinar ou esvaziar a bexiga)?	3-6 □	7-10 □	11-14 □	15-19 □	20 ou mais □
2. Quantas vezes vai ao banheiro **DURANTE A NOITE** (para urinar ou esvaziar a bexiga)?	□ 0	□ 1	□ 2	□ 3	□ 4 ou mais
3. Quando você se levanta durante a noite para esvaziar a bexiga, isso a incomoda?	□ Nunc a	□ Pouc o	□ Moderadamen te	□ Intensamen te	
4. É sexualmente ativa? □Sim □ Não					
5. Caso seja sexualmente ativa, tem ou já teve dor ou sintomas durante ou após a relação sexual?	□ Nunca	□ De vez em quando	□ Geralmente	□ Sempre	
6. Caso sinta dor na relação sexual, isso a faz evitar ter relações?	□ Nunca	□ De vez em quando	□ Geralmente	□ Sempre	
7. Sente dor associada à bexiga ou pelve (abdome inferior, lábios, vagina, uretra, períneo)?	□ Nunca	□ De vez em quando	□ Geralmente	□ Sempre	
8. Sente urgência após esvaziar a bexiga?	□ Nunca	□ De vez em quando	□ Geralmente	□ Sempre	
9. Quando sente dor, ela é geralmente	□ Nunca	□ Leve	□ Moderada	□ Intensa	
10. Sua dor a incomoda?	□ Nunca	□ De vez em quando	□ Geralmente	□ Sempre	
11. Quando você sente urgência, ela é geralmente		□ Leve	□ Moderada	□ Intensa	
12. Sua urgência a incomoda?	□ Nunca	□ De vez em quando	□ Geralmente	□ Sempre	

Se atribuído sexo <u>MASCULINO</u> ao nascimento, complete o Índice de Sintomas de Prostatite Crônica (NIH):

**1. Na última semana, sentiu qualquer dor ou desconforto nas áreas abaixo?**	
a. Área entre o reto e os testículos (períneo)	□ 1 Sim □ 2 Não
b. Testículos	□ 1 Sim □ 2 Não
c. Extremidade do pênis (sem relação com o ato de urinar)	□ 1 Sim □ 2 Não
d. Abaixo da cintura, na região púbica ou da bexiga	□ 1 Sim □ 2 Não
**2. Na última semana, sentiu:**	

 © 2023. Federação Brasileira de Ginecologia e Obstetrícia. All rights reserved.

a. Dor ou ardor ao urinar?	□ 1 Sim □ 2 Não
b. Dor ou desconforto durante ou após o clímax sexual (ejaculação)?	□ 1 Sim □ 2 Não
3. Com que frequência senti dor ou desconforto em qualquer dessas áreas (a – d, na pergunta 1) na última semana?	□ 0 Nunca<br>□ 1 Raramente<br>□ 2 Às vezes<br>□ 3 Com frequência<br>□ 4 Geralmente<br>□ 5 Sempre
4. Qual o número que melhor descreve seu nível de dor, EM MÉDIA, nos dias em que sentiu dor ou desconforto na última semana?	Sem dor Pior dor possível<br>□ 0 □ 1 □ 2 □ 3 □ 4 □ 5 □ 6 □ 7 □ 8 □ 9 □ 10
5. Na última semana, com que frequência teve a sensação de não ter esvaziado completamente a bexiga após terminar de urinar?	□ 0 Nenhuma vez<br>□ 1 menos de 1 vez em 5<br>□ 2 Menos da metade das vezes<br>□ 3 Aproximadamente metade das vezes<br>□ 4 Mais da metade das vezes<br>□ 5 Quase sempre
6. Na última semana, com que frequência precisou urinar novamente, menos de duas horas depois de ter urinado?	□ 0 Nenhuma vez<br>□ 1 menos de 1 vez em 5<br>□ 2 Menos da metade das vezes<br>□ 3 Aproximadamente metade das vezes<br>□ 4 Mais da metade das vezes<br>□ 5 Quase sempre
7. Na última semana, quanto seus sintomas o impediram de fazer as coisas que costuma fazer?	□ 0 Nenhuma vez<br>□ 1 Só um pouco<br>□ 2 Um pouco<br>□ 3 Muito
8. Quanto pensou nos seus sintomas na última semana?	□ 0 Nenhuma vez<br>□ 1 Só um pouco<br>□ 2 Um pouco<br>□ 3 Muito
8. Se tivesse que passar o resto da vida com seus sintomas como eles se apresentaram durante a última semana, como se sentiria?	□ 0 Muito feliz<br>□ 1 Satisfeito<br>□ 2 Predominantemente satisfeito<br>□ 3 Meio-termo (igualmente satisfeito e insatisfeito)<br>□ 4 Predominantemente insatisfeito<br>□ 5 Infeliz<br>□ 6 Péssimo
**Pontuação**	
**Dor**: Total dos itens 1a, 1b, 1c, 1d, 2a, 2b, 3 e 4 =	
**Sintomas urinários**: Total de vezes 5 e 6 =	
**Impacto na qualidade de vida**:Total de vezes 7, 8 e 9 =	

 © 2023. Federação Brasileira de Ginecologia e Obstetrícia. All rights reserved.

1. Histórico Psicossocial

Qual a principal fonte de estresse em sua vida?

□ Trabalho □ Família □ Financeiro □ Social □ Relacionamentos

Com quem você fala sobre sua dor em momentos de estresse?

□ Cônjuge/Companheiro(a) □Parente □ Grupo de apoio □ Religioso □ Médico/enfermeiro
□ Amigo(a) □ Profissional de Saúde Mental □ Cuido de mim mesmo(a)

Sofreu abuso ou trauma na infância (13 anos ou menos)? (Selecionar <u>todas</u> as opções aplicáveis)

□ Emocional □ Físico □ Sexual □ Violência doméstica

Já sofreu abuso como adulto?

□ Emocional □ Físico □ Sexual □ Violência doméstica

Está sofrendo abuso no momento?

□ Emocional □ Físico □ Sexual □ Violência doméstica

Já recebeu algum tratamento para saúde mental?

□ Medicamentos □ Terapia □ Hospitalização

Está recebendo algum tratamento para saúde mental? □ Sim □ Não

Em caso afirmativo, explique:

Tem história de?

□ Depressão □ Ansiedade □ Crises de pânico □ Transtorno bipolar
□Trauma □ Transtorno do estresse pós-traumático (TEPT)
□ Transtorno alimentar □ Nenhum desses

Comparado a outros fatores de estresse na sua vida, qual a importância da dor?

□ O mais importante □ Um entre muitos outros problemas

Existem relacionamentos que possam estar contribuindo para seus sintomas?

□ Sim □ Não

As pessoas que fazem parte do seu dia-a-dia te compreendem?

□ Sim □ Não

Caso você tenha um companheiro(a), descreveria como alguém que lhe dá apoio?

□ Sim □ Não

Seu(sua) companheiro(a) percebe quando você está com dor?

 © 2023. Federação Brasileira de Ginecologia e Obstetrícia. All rights reserved.

□ Sim □ Não

Como seu(sua) companheiro(a) reage quando você está com dor?

Explique:

Você acha que sua dor afeta outras áreas de sua vida?

□ Educação □ Família □ Atividades de lazer

□ Trabalho □Amigos □ Intimidade sexual

Leia cada frase e circule um número 0, 1, 2 ou 3, que indique o quanto a frase se aplicou a você na última semana. Não existem respostas certas ou erradas, não dedique muito tempo a nenhuma das frases.

DASS-21	De forma nenhuma	Parte do tempo	Boa parte do tempo	Na maior parte do tempo
Tive dificuldade de desligar	□ 0	□ 1	□ 2	□ 3
Notei secura na boca	□ 0	□ 1	□ 2	□ 3
Não tive nenhum sentimento positivo	□ 0	□ 1	□ 2	□ 3
Tive dificuldade respiratória (ex: respiração muito acelerada, falta de ar sem relação com esforço físico)	□ 0	□ 1	□ 2	□ 3
Tive dificuldade de tomar a iniciativa para fazer coisas	□ 0	□ 1	□ 2	□ 3
Tive tendência reagir exageradamente às situações	□ 0	□ 1	□ 2	□ 3
Tive tremores (nas mãos, por exemplo)	□ 0	□ 1	□ 2	□ 3
Senti que estava despendendo muita energia nervosa	□ 0	□ 1	□ 2	□ 3
Fiquei preocupado(a) com situações em que pudesse entrar em pânico e fazer papel de idiota	□ 0	□ 1	□ 2	□ 3
Senti sem perspectivas	□ 0	□ 1	□ 2	□ 3
Fiquei agitado(a)	□ 0	□ 1	□ 2	□ 3
Tive dificuldade de relaxar	□ 0	□ 1	□ 2	□ 3
Senti desanimado(a) e depressivo(a)	□ 0	□ 1	□ 2	□ 3

 © 2023. Federação Brasileira de Ginecologia e Obstetrícia. All rights reserved.

Reagi com intolerância a tudo que me impediu de seguir adiante com o que estava fazendo	□ 0	□ 1	□ 2	□ 3
Senti a ponto de entrar em pânico	□ 0	□ 1	□ 2	□ 3
Nada me entusiasmou	□ 0	□ 1	□ 2	□ 3
Senti desvalorizado(a) como pessoa	□ 0	□ 1	□ 2	□ 3
Fiquei excessivamente sensível	□ 0	□ 1	□ 2	□ 3
Percebi o trabalho do meu coração na ausência de esforço físico (ex: sensação de elevação da frequência cardíaca, falha de batimentos)	□ 0	□ 1	□ 2	□ 3
Tive medo sem motivo justificável	□ 0	□ 1	□ 2	□ 3

<u>ESTÁ USANDO</u> atualmente, ou usou alguma das substâncias abaixo nos <u>ÚLTIMOS 12 MESES</u>? (Selecionar <u>todas</u> as opções aplicáveis)

Substância			Quantas vezes por semana?			Usa isso para controle da dor?	
Alguma bebida alcoólica	□ Não	□ Sim	□ <1	□ 2-3	□ >4	□ Sim	□ Não
Tabaco ou produtos que contêm nicotina	□ Não	□ Sim	□ <1	□ 2-3	□ >4	□ Sim	□ Não
Cocaína / Crack	□ Não	□ Sim	□ <1	□ 2-3	□ >4	□ Sim	□ Não
Heroína	□ Não	□ Sim	□ <1	□ 2-3	□ >4	□ Sim	□ Não
Opioides	□ Não	□ Sim	□ <1	□ 2-3	□ >4	□ Sim	□ Não
Metanfetaminas	□ Não	□ Sim	□ <1	□ 2-3	□ >4	□ Sim	□ Não
Estimulantes	□ Não	□ Sim	□ <1	□ 2-3	□ >4	□ Sim	□ Não
Ecstasy	□ Não	□ Sim	□ <1	□ 2-3	□ >4	□ Sim	□ Não
Psicodélicos	□ Não	□ Sim	□ <1	□ 2-3	□ >4	□ Sim	□ Não
Maconha/THC/Cannabis	□ Não	□ Sim	□ <1	□ 2-3	□ >4	□ Sim	□ Não

 © 2023. Federação Brasileira de Ginecologia e Obstetrícia. All rights reserved.

Obrigado por dedicar seu tempo ao preenchimento deste formulário. Essas informações ajudarão os profissionais de saúde a cuidar melhor de você.

Para mais informações sobre dor pélvica crônica, e como se preparar para a avaliação clínica, visite as sessões de "recursos do paciente" e "panfletos" do site da *International Pelvic Pain Society* [Sociedade Internacional de Dor Pélvica], em www.pelvicpain.org.

Exclusivamente para uso oficial:

- Formulário revisado por (nome):
- Data da revisão:
- Comentários do profissional de saúde:

 © 2023. Federação Brasileira de Ginecologia e Obstetrícia. All rights reserved.
